# HBV induces inhibitory FcRL receptor on B cells and dysregulates B cell-T follicular helper cell axis

**DOI:** 10.1038/s41598-018-33719-x

**Published:** 2018-10-17

**Authors:** Bhawna Poonia, Natarajan Ayithan, Madhuparna Nandi, Henry Masur, Shyam Kottilil

**Affiliations:** 1 Division of Clinical Care and Research, Institute of Human Virology, University of Maryland School of Medicine, Baltimore, MD 21201 USA; 2 Critical Care Medicine Department, NIH Clinical Center, Bethesda, MD 20892 USA

## Abstract

Spontaneous or treatment induced seroconversion in chronic HBV infection is rare and generation of anti-HBs antibodies is the current goal of HBV therapeutics. Here we investigated B and follicular T helper (Tfh) cell defects that persist in HBV infection despite long-term nucleos(t)ide analog (NUC) treatment and possible mechanisms behind them. RNA sequencing revealed that patient B cells have upregulated expression of multiple inhibitory receptors including members of FcRL family and downregulation of genes involved in antigen presentation. An expansion of atypical memory CD19^+^CD10^−^CD27^−^CD21^−^ subset of B cells, that express high levels of FcRL5, is persistently present in patients. HBs antigen specific IgG response is concentrated in classical memory and not in atypical memory subset, confirming dysfunction of this subset. Activated Tfh, which expressed excessive CD40L upon polyclonal stimulation, were present in patients. Incubation of B cells from healthy individuals with HBV core (HBc) or CD40L resulted in induction of inhibitory receptors FcRL4, FcRL5 and PD-1 on CD19+ cells and resulted in altered B cell phenotypes. Mechanistically, HBc binds B cells and causes proliferation specifically of FcRL5+ B cell subset. Our results provide evidence that HBV directly causes upregulation of inhibitory pathways in B cells resulting in an accumulation of atypical B cells that lack anti-HBs function.

## Introduction

Chronic HBV (CHB) infection is incurable with currently available nucleoside analog therapies that can best provide efficient virus suppression with life-long use. The clinical measure of functional cure, loss of HBV surface antigen (HBs) and generation of anti-HBs antibodies, is an extremely rare outcome of these therapies^1,2^. Effective CD4 T cell, CD8 T cell and B cell responses are established during resolution of an acute infection^3^, while such coordinated immune response is absent during persistent infection even with long-term treatment. Although generation of anti-Hbs antibodies in chronic patients and their circulation in form of antigen bound immune complexes has been long been shown^4^, free antibodies are not detectable and it is clear that these antibodies are not produced in quantities required to neutralize antigen sufficiently. Importance of neutralizing antibodies in vaccine-induced protection is well known and increasingly evidence suggests antiviral activity of neutralizing antibodies may have clinical implications for treatment of chronic infection^5,6^. This observed lack of seroconversion to anti-HBs antibodies even with long-term antiviral treatment with nucleos(t)ide analogs (NUC) points to persistent defects in humoral compartment that are not completely reversed with suppression of virus replication^1,7^.

For effective B cell response, several signals need to be delivered to an antigen specific B cells. These are antigen recognition and binding by BCR, optimal signaling between helper T cells and B cells as well as Toll-Like Receptor (TLR) signaling^8,9^. T cell help to B cells is provided in the form of expression of molecules like CD40L and IL-21, which promote B cell proliferation and survival. Since antiviral therapies fail to achieve sustained response (defined by HBsAg loss and seroconversion and absent plasma HBV DNA) in most patients, it is important to investigate B cell and follicular T helper cell defects in patients and study if these defects improve with virus suppression. B cell activation is observed in chronic HBV infection^10,11^. Recently, in immune active patients, a reversal of B cell hyperactivation was shown to be associated with HBsAg seroconverion^11^. Moreover, this activation was positively correlated with CD40L levels in the serum. Chronic patients also have lower levels of memory B cells as well as show downregulation of co-stimulatory molecules, defects that are reversed in patients that resolve their infection^11^. These studies hint at Tfh-B cell abnormalities that hamper HBsAg loss and seroconversion in most CHB patients.

It is not well established whether HBV induced B cell defects are resolved with antiviral treatment. Other chronic infections have persistent defects in B cell phenotypes and function that may or may not be corrected with effective virus control. During chronic HIV infection, abnormal expansion of CD19+ CD10−CD20+ CD27−CD21− tissue like memory B cells expressing inhibitory receptor FcRL4 occurs, although this expansion is normalized with effective antiretroviral therapy^12^. Similar increase in these so called exhausted B cells occur during chronic hepatitis C infection^13^, cure of HCV using DAA therapy does not however normalize these expanded cells^14^ (and our unpublished data). Plasmodium falciparum infection results in similar increase in these defective B cells with impaired B cell receptor signaling and responses^15^.

Here we characterized B and Tfh cells during chronic HBV infection and sought to understand the effect of long-term virus suppression with NUC on their phenotypes and functions. Our results reveal that HBV infection results in an increased expression of multiple inhibitory receptors on B cells along with expansion of dysfunctional B cells which persists with 80–90 weeks of NUC therapy. Mechanistically, both HBV antigens and CD40-CD40L interaction play a role in generation of abnormal B lymphocytes in CHB.

## Results

### B cells from CHB patients have distinct transcriptome profile characterized by inhibitory receptors

**Figure 1:**
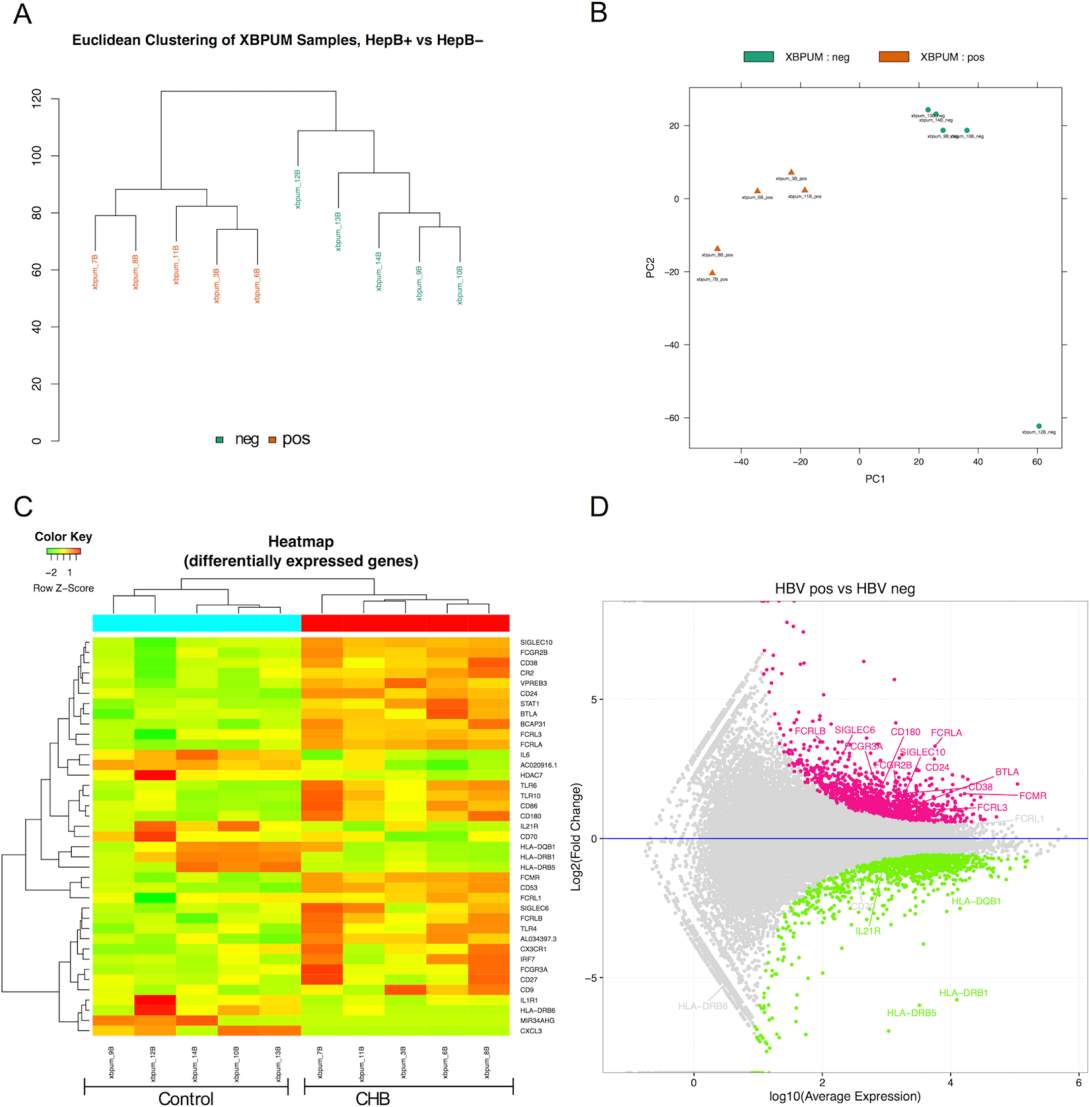
Inhibitory Fc receptor gene upregulation in B cells during CHB revealed by RNA sequencing. Transcriptome analysis of CD19+ sorted peripheral blood lymphocytes of CHB (HepB+ or pos) and controls patients (HepB− or neg) (n = 5 each). (**A**) Euclidian distance illustrates hierarchical clustering of the groups. (**B**) Principal component analysis differentiates between HBV+ and HBV− conditions and shows variance amongst replicates. (**C**) Heat map of subset of differentially expressed genes (FDR corrected p < 0.05) in B cells from CHB and control patients. (**D**) MA scatter plot shows upregulated (pink) and downregulation (green) genes from FcRL and HLA-DR families from CHB (HBV pos) relative to control (HBV neg) B cells.

Our first goal was to identify signatures of global B cell dysfunction in CHB that can point us to possible mechanisms of B cell abnormalities during persistent infection. We approached this by comparing transcriptome of CD19+ cells, that includes all B cell subsets. The composition of B cells in CHB and healthy controls with respect to proportions of naïve or memory subsets is likely to differ and the gene expression signature is expected to reflect this difference. We used RNA sequencing to profile sorted CD19+ cells. Overall, there were 939 up-regulated and 968 down-regulated genes in CHB positive CD19+ B cell samples relative to controls. Euclidean clustering and principal-component analysis of filtered data showed a clear segregation of the control and CHB groups (Fig. 1A,B). Genes upregulated from CHB patients included several members of inhibitory pathways of the Fc family receptors including FCGR2B, FcRLA, FcRLB, FcRL1, FcRL3. FcRL5, which identifies dysfunctional atypical memory B cells^16^, showed modest 1.5-fold up-regulation in B cells from patients with an FDR value of 0.34 > FDR cut-off of 0.05. Markers of immature/transitional B cells (CD38, CD24) were also highly expressed in CHB B cells. Among the downregulated genes were those involved in B cell development, antigen presentation, BCR signaling (e.g., HLA-DR family, CD70, IL-21, IL-6) (Fig. 1C,D). Ingenuity Pathway enrichment analysis also revealed differences in expression of genes from pathways involved in B cell development, BCR signaling, Interferon signaling and TLR signaling. Complete list of DEGs and top canonical pathways are shown in Supplementary Tables 1 and 2.

The dysfunctional atypical memory B cell subset in malaria and HIV infections shows upregulated expression of inhibitory genes including FcRL5, FcRL3, FcGR2B, LILRB2 and Siglec6^17^, all of which were upregulated in total B cell from our CHB patients; this possibly reflects an expansion of atypical B cell subset in CHB patients. Next, we profiled B cells to identify phenotypes and functions that these gene signatures indicate.

### Atypical memory B cells persist in CHB patients despite long-term adefovir treatment

**Figure 2:**
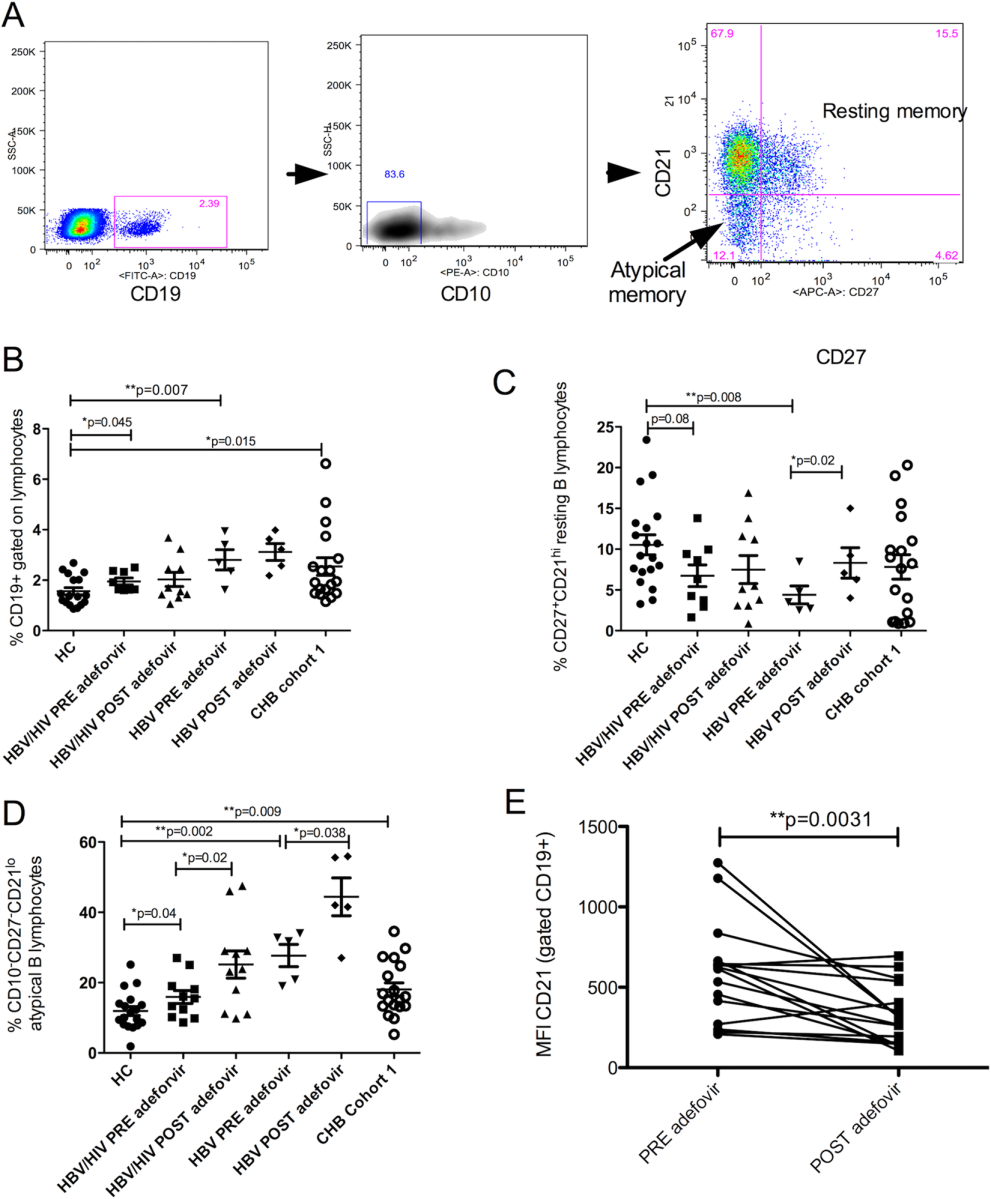
Expanded atypical memory B cells in chronic HBV infection. (**A**) CD19+ B cells were gated on live CD3− lymphocytes and gates for further evaluating resting and atypical memory B cells are shown. Frequency of total CD19+ lymphocytes (**B**) and of resting memory B lymphocytes (**C**) in samples from before and after Adefovir treatment in e-antigen positive HBV/HIV (n = 11) and HBV mono-infected (n = 5) groups and cohort 1 of e-antigen negative chronic HBV-infected patients (n = 18) relative to healthy controls (n = 20). (**D**) Frequency of CD27^lo/−^CD21^−^CD10^−^ atypical memory B lymphocytes before and after Adefovir treatment in HBV/HIV (n = 11) and HBV mono-infected (n = 5) groups and cohort 1 of chronic HBV-infected patients (n = 18) relative to healthy controls (n = 20). (E) Significant downregulation in CD21 MFI in paired samples post-Adefovir treatment from HBV/HIV and HBV groups combined (n = 16). Differences between the groups were analyzed by Wilcoxon test. Before and after treatment samples were analyzed by paired t-test. *Indicates significant difference.

HBV infection resulted in increase in frequency of circulating CD19^+^ lymphocytes in all cohorts of chronic HBV patients (Fig. 2A,B). The resting memory B cells (CD21^+^CD27^+^) were reduced in patients, and this subset increased with long-term therapy to normal levels (Fig. 2C). Deep sequencing of IgH B cell receptors revealed increased sum of productive frequency of top 10 IgH rearrangements post treatment relative to frequencies in paired sample from baseline (Supplementary Fig. 1: patient#1: increase from 0.31% to 0.9%; patient#2: increase from 0.3% to 0.44%); an increased clonality of certain BCR sequences post therapy, reflecting increase in memory population. Increased frequency of CD21^lo^/^−^CD27^−^ atypical memory B cells was present in all chronic HBV patient groups relative to healthy controls despite varying levels of viremia and e-antigen status of these cohorts. There was no normalization of this subset of B cells with treatment (Fig. 2D,E) and in fact, we observed a significant downregulation of CD21 and consequent increase in CD21^lo^/^−^CD27^−^ subset after long term NUC therapy (Fig. 2D,E). Paired peripheral and liver lymphocyte analysis from 3 patients showed liver had significantly higher frequency of this subset relative to PBMC (PBMC vs LIL Mean ± SD 26.04 ± 6.8 vs 58.6 ± 15.88, p < 0.003). Since we did not have access to healthy liver, we cannot conclude whether these atypical cells in liver are expanded due to CHB presence or if it is a normal liver B cell phenotype.

Thus, the observation of increase in atypical B cell frequencies in CHB is consistent with increased expression of genes of inhibitory pathways including FcRL that are implicated in generation of these cells.

### Anti-HBs specific B cells are located in classical and not in atypical memory B cell compartment

**Figure 3:**
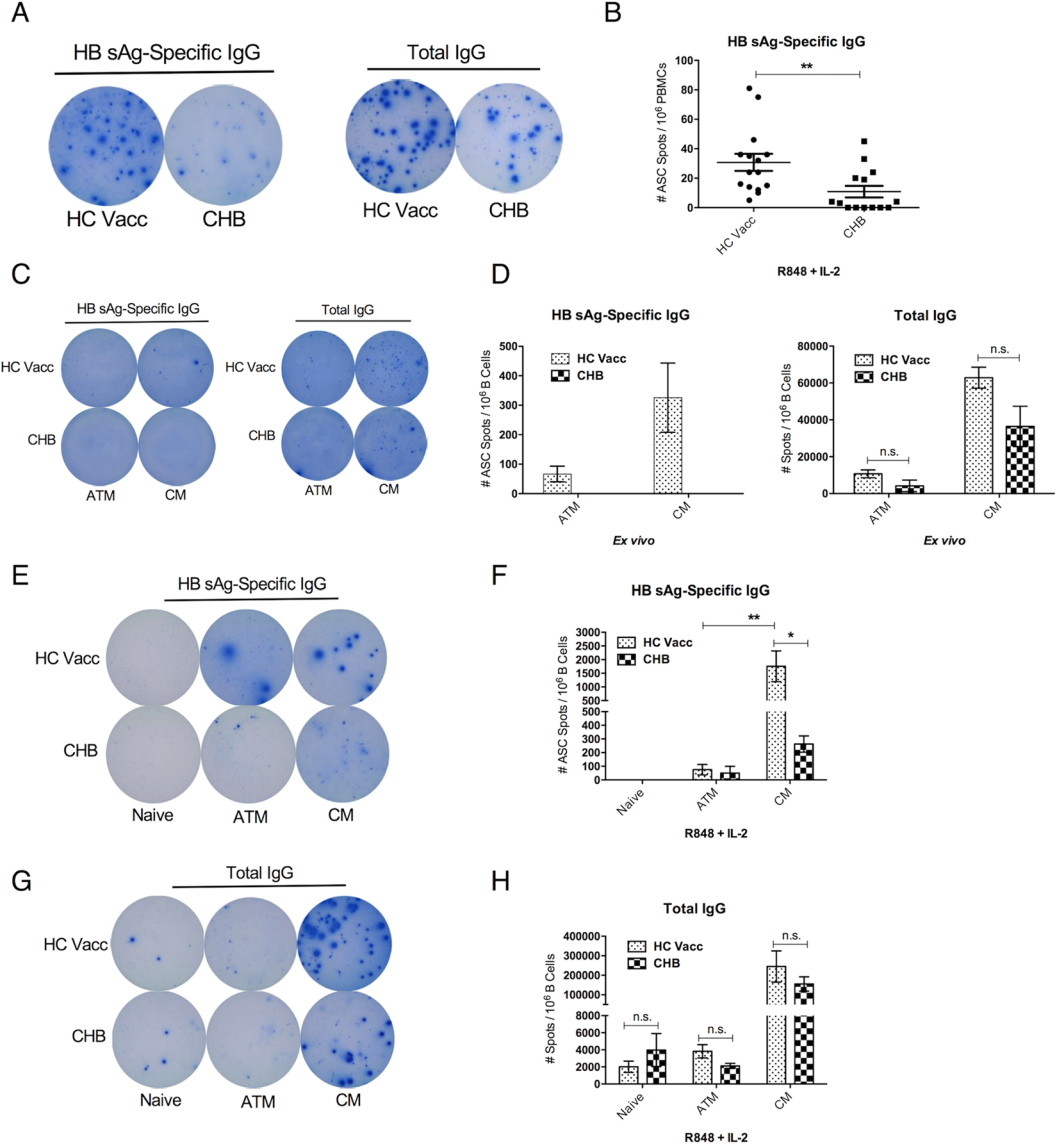
HBs antigen specific B cells are located in classical memory compartment. Representative B cell Elispot data (**A**) and individual ASC spots (**B**) in total PBMCs from healthy HBV vaccinated (HC vacc) (N = 15) or CHB patients (N = 15) that were cultured for 5 days in presence of R848 + IL-2 show HBsAg-specific IgG and total IgG responses. Representative (**C**) and average (**D**) HBs specific and total IgG response in sorted classical memory and ATM B cell subsets from HC vacc (N = 3) and CHB patients (N = 3) *ex vivo*. Samples from HC vacc are tested 7 days after last booster injection. Representative (**E**) and average (**F**) HBs antigen specific and total (G-H) IgG secreting B cells among sorted naive, ATM and CM B cell subsets from HC vacc (N = 3) and CHB patients (N = 3) after culture in presence of R848 + IL-2. Paired and unpaired two-tailed t-tests were done. *(p ≤ 0.05), **(p ≤ 0.005) represents statistical significant differences.

B cell ELISPOT was used to measure HBs antigen specific B cell response in CHB patients. We optimized procedure for stimulation of memory B cells. R848 + IL-2 combination, which is described by others as well^18,19^ was found superior compared with other stimuli tested, including PWM and CpG. Compared with individuals with a history of HBV vaccination, CHB patients had significantly lower number of spots positive for HBs specific response (Fig. 3A). We then evaluated compartmentalized HBs specific B cell response in sorted atypical memory, classical memory and naïve B cells subsets. As expected, in recently HBV vaccinated individuals, memory B cells (CD19+ CD27+) contained HBs specific B cells *ex vivo*, which likely reflects presence of plasma cells in these individuals (samples tested were collected one week after last HBV vaccine booster). No HBs specific B cells were detected *ex vivo* from CHB patients (Fig. 3A). Upon culturing cells in presence of R848 to generate memory B cells, highest level of HBs specific response was concentrated in classical memory B cells, whereas significantly lower HBs specific B cell response was detected in atypical memory compartment (Fig. 3A,B). Also, significantly lower HBs specific B cells were present in classical memory subset from CHB patients relative to vaccinated individuals (Fig. 3C), as expected.

Thus, atypical memory B cells are dysfunctional in generating HBs specific antibody response and their accumulation in CHB at the expense of classical memory B cells is likely to compromise antigen specific response in patients.

### Increased activated Tfh in CHB patients

**Figure 4:**
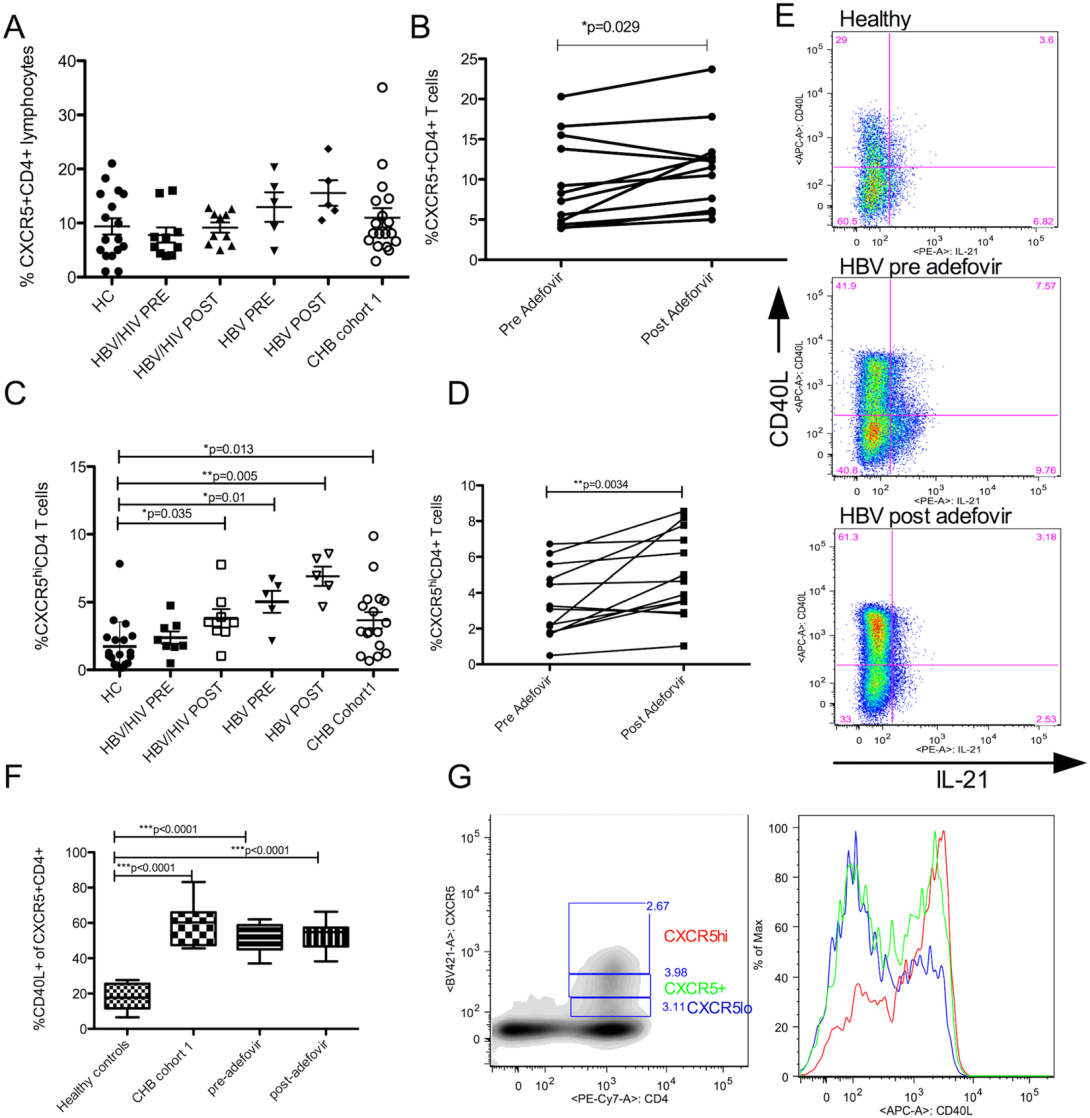
Activated Tfh expand during chronic HBV infection. (**A**) Frequency of CXCR5+ CD4+ Tfh and (**C**) CXCR5hiCD4+ subset in samples from pre and post Adefovir treatment in e-antigen positive HBV/HIV (n = 11) and HBV mono-infected (n = 5) groups and cohort 1 of e-antigen negative chronic HBV-infected patients (n = 18) relative to healthy controls (n = 20). Change in CXCR5+ CD4+cell frequency (**B**) or in CXCR5hiCD4+ cell frequency (**D**) with Adefovir treatment in paired samples from HBV/HIV (11) or HBV (n = 5) patients. (**E**,**F**) CD40L expression in CXCR5+ Tfh from chronic HBV patients from cohort 1 (n = 15) and before and after therapy combined (n = 15) relative to controls (n = 15). (**G**) CD40L expression among different Tfh subsets defined by CXCR5 expression levels (CXCR5hi, CXCR5+, CXCR5lo shown by density plot gates). Differences between the groups were analyzed by Wilcoxon test. Before and after treatment samples were analyzed by paired t-test. *Indicates significant difference.

To test whether B cell abnormalities are linked to follicular helper T cell defects, we profiled these cells using flow cytometry. While the frequency of CXCR5^+^CD4^+^ T cells trended lower (p = 0.06) in HBV patients relative to healthy controls, interestingly the frequency of CXCR5^hi^CD4 Tfh was significantly higher in patients (p < 0.02) (Fig. 4A–C). Total CXCR5^+^CD4^+^ Tfh frequency increased in post treatment samples in paired analysis (p < 0.02) (Fig. 4B); while expanded CXCR5^hi^CD4^+^ Tfh did not normalize with treatment. CHB patients had significantly increased frequency of terminally differentiated (CCR7−CD45RA+) Tfh relative to controls (mean ± SD 2.56 ± 3.2 vs 25.74 ± 15.01, p < 0.001). These results indicate a persistently activated Tfh profile despite treatment of CHB infection.

In response to PMA/I stimulation, CXCR5^+^CD4^+^ T cells from patients upregulated CD40L at significantly higher levels compared with healthy controls (P < 0.0001) (Fig. 4E,F), suggesting these cells are hyperactivated. The Tfh subsets distinguished by levels of CXCR5 expression (CXCR5hi, CXCR5+ and CXCR5lo) had CD40L expression that corresponded with CXCR5 expression levels of the subset (Fig. 4G). There was no difference in IL-21 production from these cells from patients and controls (%IL-21^+^ Tfh mean ± SD from HBV vs healthy were 18.23 ± 8.01 and 17.25 ± 10.27, respectively).

### HBV proteins and CD40L upregulate inhibitory receptors on B cells

Since atypical B cells are regarded as “exhausted” B cells^17^, a possible mechanism behind their expansion could be continuous triggering of BCR, as is the case for exhausted T cells during chronic infections^20^. Additionally, our finding of excessive CD40L expression from patient Tfh prompted us to investigate the consequence of continuous BCR and CD40L signaling on altering B cell phenotype. BCR stimulation with F(ab’)2 for 4 days resulted in significant increase in the atypical memory (CD21^−^CD27^−^) subset, while CD40L stimulation caused an expansion of the CD21^+^ naïve subset (Supplementary Fig. 2). There was no significant alteration in these subsets between day 0 and day 4 untreated cultures, indicating culture alone does not induce these subsets (data not shown). It is plausible that during CHB infection antigen presence along with excessive CD40L produced by Tfh results in altered B cell differentiation and accumulation of more naïve and atypical memory B cells.

**Figure 5:**
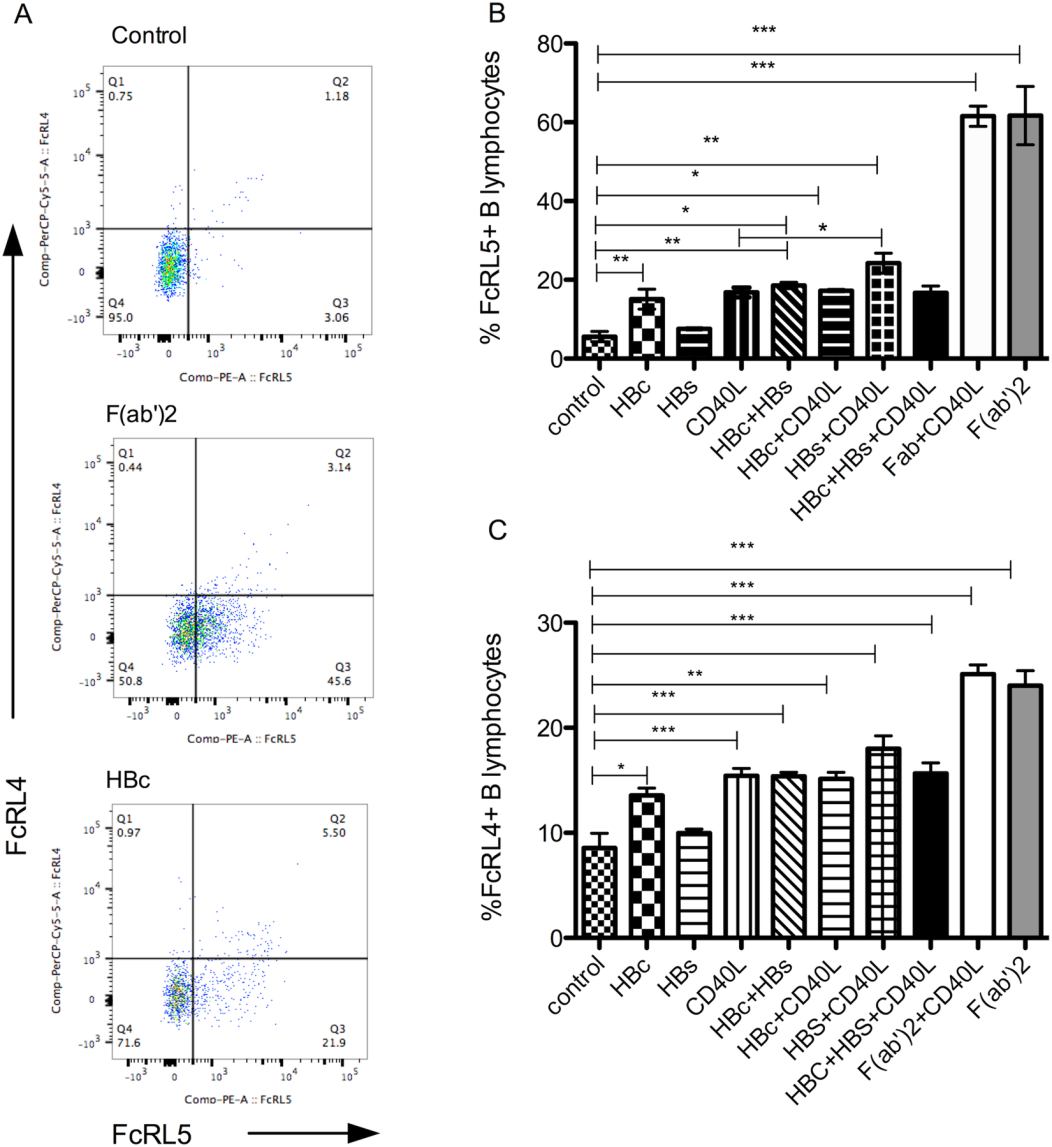
HBV core protein upregulates FcRL inhibitory receptors on peripheral B lymphocytes. (**A**) FcRL4 and FcRL5 expression on CD19+ lymphocytes assessed after 4 days incubation of PBMC with F(ab’)2 or HBV core protein. (**B**) Average FcRL5 expressing CD19+ lymphocyte frequencies upon incubation of PBMC with combinations of HBV proteins, CD40L or F(ab’)2. (**C**) Average FcRL4 expressing CD19+ lymphocyte frequencies upon incubation of PBMC with combinations of HBV proteins, CD40L or F(ab’)2. Each bar represents average of five different samples tested in two independent experiments. Wilcoxon signed rank test for paired samples (*p < 0.02, **p < 0.001, ***p < 0.0001).

Our transcriptome data from B cells of CHB patients revealed enrichment of genes in the Fc family of inhibitory receptors (Fig. 1). CHB patient B cells had higher cell surface expression of FcRL5 relative to healthy controls (mean 10.5% vs 4.7%), also expression of FCRL5 and FCRL4 was significantly higher on atypical memory B cells (mean 26.2%) compared with naïve (mean 2.28%) or classical memory B cells (mean 11.16%) (Supplementary Fig. 3). FcRL5 is a known key factor in generation of atypical memory B cells^16^. We tested whether HBV proteins directly cause phenotypic alterations in B cells. HBV core protein (HBc) is known to be highly immunogenic and results in activation of naïve B cells^21^. PBMC from healthy individuals were incubated with combinations of HBc, HBs, F(ab’)2 and CD40L for 3 days and resulting phenotypes were tested with flowcytometry. Interestingly, HBc incubation resulted in significant upregulation of inhibitory receptors FCRL4 and FCRL5 on CD19+ B cells (p < 0.01) (Fig. 5A–C) and there was a dose dependency in this FcRL upregulation (Supplementary Fig. 4). BCR (F(ab’)2) or CD40L ligation also upregulated these receptors on B cells. HBsAg incubation by itself did not alter expression of these receptors but enhanced the effect of CD40L (Fig. 5B,C).

**Figure 6:**
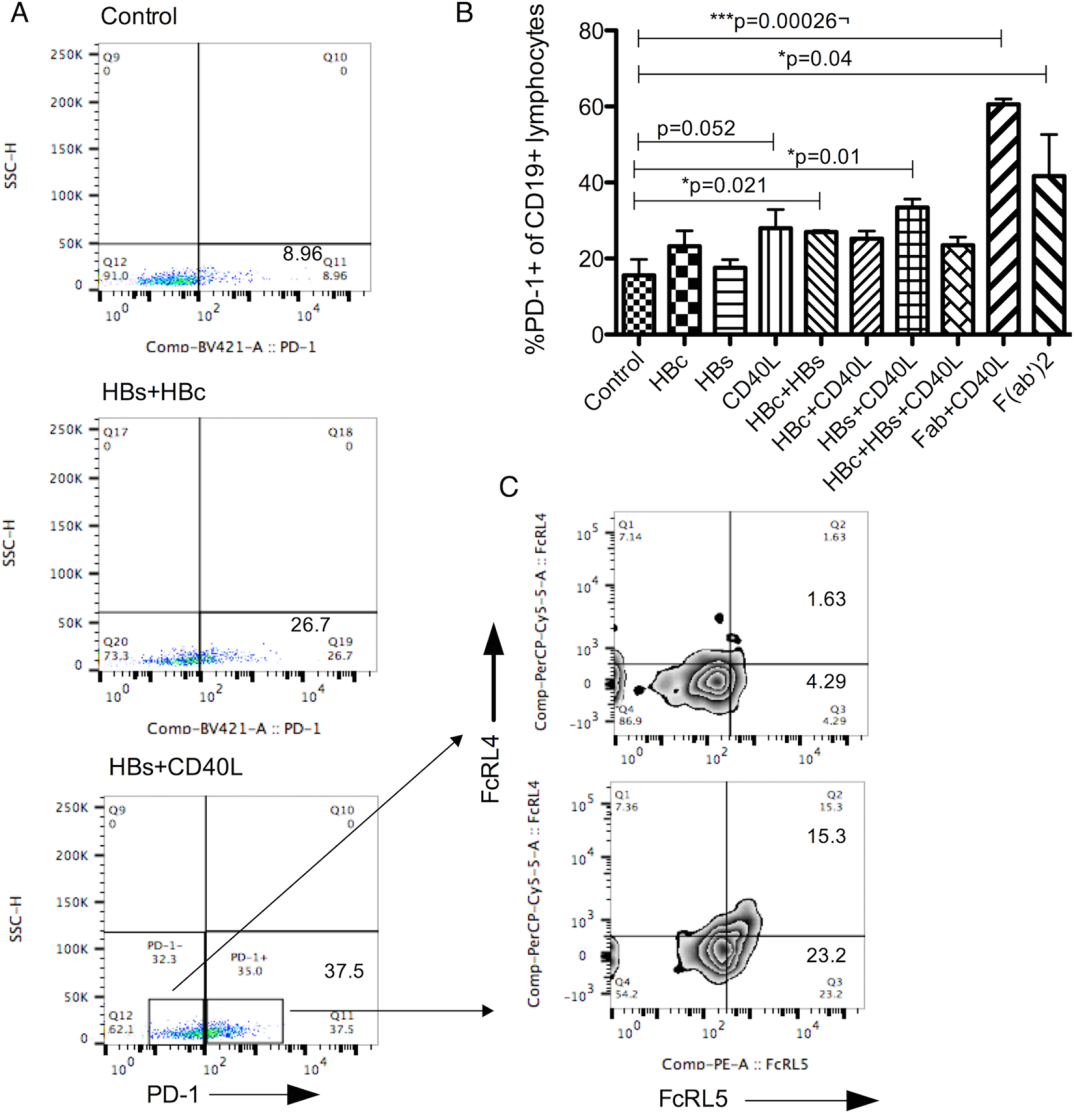
HBV proteins upregulate PD-1 on peripheral B lymphocytes. (**A**) PD-1 expression measured on CD19 gated lymphocytes after incubation of cells with HBV proteins or CD40L. (B) F(ab’)2, CD40L and HBV protein combinations were used to stimulate PBMC and PD-1 expression measured in CD19+ lymphocytes. (C) Expression of FcRL4 and FcRL5 on PD-1+ or PD-1- gated lymphocytes. Each bar represents average of five different samples tested in two independent experiments. Wilcoxon signed rank test for paired samples p < 0.05 considered significant. (*p < 0.02, **p < 0.001, ***p < 0.0001).

Similar to effect on FCRL expression, F(ab’)2, CD40L and HBV antigen mediated signaling also resulted in an increase in PD-1 expression on CD19^+^ cells (Fig. 6A,B). These PD-1^+^ B cells had significantly high levels of FcRL5 compared with PD-1^−^ subset from same samples (Fig. 6C) (p < 0.001), which is the phenotype of exhausted B cells. Next, we sought to define mechanisms by which HBV proteins potentially result in generation of observed abnormal B cell phenotypes in our patients.

### HBV core antigen binds B cells and affects B cell proliferation

**Figure 7:**
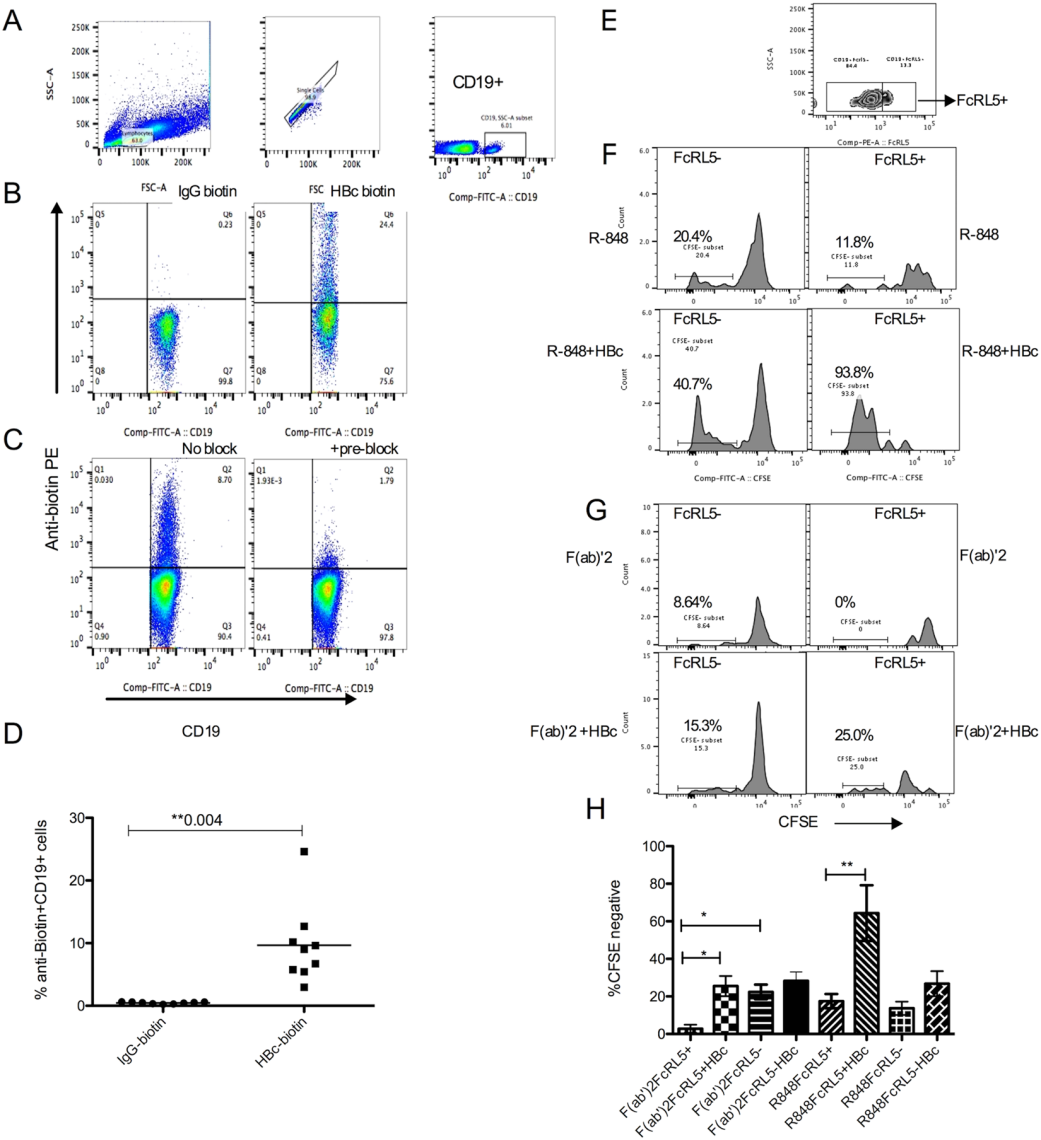
HBV core protein binds CD19+ lymphocytes and alters proliferation. (**A**,**B**) Strategy for detection of HBVcore biotin labeled CD19+ cells with anti-biotin PE secondary antibody is shown. CD19+ lymphocytes incubated with IgG biotin detected with anti-biotin PE serve as control. (**C**) Comparison of anti-biotin PE+ cells detectable without and with pre-blocking of cells with unlabeled HBc protein shows specificity of HBc binding. (**D**) Average frequency of HBVcore+ CD19+ lymphocytes detected in samples from nine different donors in four independent experiments is shown. (**E**) Gating for FcRL5+/− CD19+ lymphocytes. (**F**–**H**) Proliferation of CFSE labeled FcRL5+ or FcRL5− B cells in presence of HBcore. CFSE dilution in representative R-848 (**F**) or F(ab)′2 (**G**) stimulated FcRL5− versus FcRL5+ gated CD19+ subsets in absence or presence of HBcore. (**H**) Average CFSE dilution from three samples show percentage of CFSE negative proliferated FcRL5+ or FcRL5− B cells in presence or absence of HBc. Paired t-test p < 0.05 considered significant.

To elucidate the mechanism of HBV core protein caused B cell defects, we hypothesized that HBc directly impairs B cells. Normal PBMC were tested for HBc binding of B lymphocytes using biotinylated HBc followed by avidin or anti-biotin secondary antibodies (Fig. 7A,B). Varying degrees of HBc binding was observed in 9 separate samples (Fig. 7C). Specificity of HBc binding to B cells was confirmed by pre-blocking cells with an unlabeled HBc that resulted in an average of 80% reduction in HBc biotin positive cells (Fig. 7D). We then tested effect of core binding on B cell proliferation by culturing CFSE labeled cells from healthy individuals in F(ab’)2 or R-848 in presence or absence of HBc antigen. FcRL5^+^ B cells had significantly lower (p < 0.05) frequency of cells that diluted CFSE relative to FcRL5^−^ B cells in 4 days cultures with either F(ab)’2 or R848, confirming the inhibitory action of FcRL on both BCR and TLR mediated B cell proliferation. Interestingly however, addition of HBc to the cultures resulted in significantly increased proliferation, specifically of the FcRL5^+^ subset (average ± stdev FcRL5+ cells that diluted CFSE was 2.7% ± 3.7 in control versus 25.5% ± 9.3 in cultures with HBcore (p < 0.01), providing a possible mechanism by which these cells accumulate during CHB infection (Fig. 7E–G).

## Discussion

Our study established that humoral defects persist during treated chronic HBV infection with a preferential expansion of atypical B cells of exhaustive phenotype and function. In this regard, we further established that B cell defects are influenced by an abnormal expansion of follicular helper T cell subsets with high CD40L expression. Furthermore, we also demonstrated that exposure to HBV core protein induces the dysfunctional profile of B cells from healthy controls establishing a novel mechanism for humoral dysfunction associated with CHB infection.

A major limitation to curing CHB infection with current NUC therapy is lack of anti-HBs antibody generation even after long-term treatment with currently available antiviral drugs. Thus, seroconversion marks a functional cure and is the goal of HBV therapeutics, which is not achievable in most patients with current antiviral therapy. However, the exact mechanism underlying a lack of effective protective humoral response in chronically infected HBV patients is not yet understood. In this study, we demonstrate an abnormal expansion of atypical B cells in CHB infection, which are defective in generating HBs specific antibody secreting cells. A similar expansion of tissue-like memory or atypical “exhausted” B cells is shown for other chronic infections including HIV, HCV and malaria^12,14,15^. Since chronic antigen exposure is a plausible factor behind their expansion, normalization of these cells is expected with effective virus control. Both early and chronic HIV infection induced expansion of TLM cells was normalized after 12 months of ART initiation and with resulting undetectable viremia^12^. Our results show no reduction in frequency of these CD21^lo^ B cells even with long-term antiviral therapy. In fact, we observed a significantly expanded atypical memory B cell subset in many patients after more than 80 weeks of therapy. One explanation could be that most of these patients had significant plasma viral loads (range 200–2000000 copies/ml; data not shown) at ~80–90 weeks treatment period; we believe that viral antigens were originally responsible for expansion of this abnormal subset and their persistence keeps these cells activated and thus exhausted. During chronic SIV infection, observations of an increase in such tissue-like memory B cells, which persist with antiretroviral therapy in infected macaques^22^, where viral suppression on ART was suboptimal, support our reasoning. For human HIV infection, an elevated tissue-like memory phenotype despite effective ART^23^ was shown recently, however those results were compared between uninfected and HIV infected individuals and don’t reflect temporal change with therapy. In another report on HIV infection of young children (under 10 years), among the low-viremia group proportions of tissue-like memory B cells were higher in ART-naive children than in ART-treated children^24^. One reason for the ongoing TLM presence and expansion in our cohort potentially is continued antigen presence despite therapy. It should be noted that HBV antigens, especially HBsAg levels stay high in NUC-treated individuals^25^ and this also provides a potential source of stimulation for B cells. Long-term Adefovir therapy is not known to cause adverse immune side effects. However, *in vitro* studies show certain nucleoside reverse transcriptase inhibitors induce mitochondrial toxicity in human B-lymphocytes and impair the immunoglobulin synthesis^26^; this possibility was not tested here. Our cohort of Adeforvir treated patients was composed of e antigen positive, high viremia individuals where, although treatment resulted in effective (>4 log) virus reduction, detectable viremia persisted. We cannot rule out the possibility that in other groups such as e-negative patients or those where undetectable viremia is achieved with currently available NUCs some recovery in B cell phenotypes might be observed, akin to observations in ART controlled HIV infection.

Another likely reason behind abnormal B cells is hyperactive Tfh cells; follicular helper T cells in HBV were activated or expanded in previous studies^27,28^. We observed specific expansion of the CXCR5^hi^ subset of Tfh from chronic patients. Moreover, upon stimulation Tfh cells expressed significantly elevated levels of CD40L from patients compared with controls, indicating an overactive phenotype. CD40 signaling in B cells is key to plasma cell formation and antibody secretion. However the strength of this interaction can tilt the differentiation pathway of B cells; higher concentrations of cell bound CD40L are shown to arrest the terminal differentiation of B cells to generate long lived plasma cells^29^. In this regard, persistence of CD40 signaling is important for inhibition of differentiation and B cell recovery is made within 24 hours of removal of signal^29^. We showed here that activated Tfh expressing CD40L persist even in treated CHB infection; this could directly impact plasma B cell generation and resulting antibody production. A limitation of the study is it’s peripheral nature. While the exhaustive immune changes in B cells were exaggerated in the liver than in peripheral blood from patients, unavailability of liver from non-CHB patients precluded detailed characterization of HBV related intrahepatic humoral immune defects. Additionally, analysis of antigen specific Tfh was also not done here. Vaccine antigen-specific Tfh can be detected in germinal center and periphery, nevertheless peripheral antigen specific Tfh detection during chronic infection is difficult^30,31^. We were not able to reliably detect HBV antigen specific cytokine production in peripheral Tfh from CHB patients. Ongoing studies explore the Tfh-B interactions in lymph nodes of patients with CHB.

RNA sequencing of B cells from CHB patients revealed higher level expression of the genes in several inhibitory pathways relative to controls. Genes in the Fc receptor like (FcRL) family are specific to B cells and their role in attenuating BCR signaling is increasingly getting recognized^32,33^. We hypothesized that HBV antigens and abnormal Tfh play a role in generation of B cells with phenotypes of dysfunctional cells. To investigate the effects of continued antigen presence as well as abnormal CD40L signaling we cultured peripheral blood cells with BCR stimulants, HBV antigens and CD40L. BCR and CD40L signaling resulted in upregulation of FCRL4 and FCRL5. Similarly, HBV antigens, especially HBc caused significant upregulation of inhibitory markers including FcRL4, FcRL5 and PD-1 on B cells. FCRL5 was identified as a key factor required for generation of atypical B cells^16,34^ whereas FCRL4 is highly expressed in B cells from HIV viremic individuals and is enriched in atypical B cells^12^. It is not known by what mechanism HBc or HBs can influence B cell signaling. For HIV infection, gp120 binds to naive B cells through α4β7 expressed on B cells and results in FcRL4 expression^35^. Whether some receptor is expressed on B cells that binds HBc or HBs is not known. However, HBc binding to B cells was demonstrated in our results as well as it is known that HBc binding to naïve human B cells resulting in their activation^21,36,37^. In humanized mouse model HBc injection resulted in activation of naïve B cells along with production of anti-HBc IgM in chimeric mice^21^. A linear motif present in the FR1-CDR1 junction of the heavy or light chain of the B-cell surface receptor was shown to bind HBc antigen on naive B cells^36^. During CHB infection, serum HBc Ag concentration is closely correlated with serum HBV DNA as well as intrahepatic cccDNA level; a reduction in HBc levels correlates with e antigen seroconversion^38,39^. In e antigen positive CHB infection or during immune tolerant/clearance phases, when HBc levels in serum are high^40^, this immunogenic antigen has potential to cause inhibitory signaling in B cells.

We propose that HBV core protein binding to B cells results in increased activation and proliferation of these cells. Moreover, specific proliferation of FcRL5+ B cells with continued HBc signaling during chronic infection potentially results in accumulation of atypical memory B cells. A similar accumulation of such atypical memory cells that persisted despite ART treatment of SIV infected macaques explained the lack of increased serum antibody levels observed upon Env-boosting of the animals^22^. We believe a similar mechanism would also prevent the lack of response to the otherwise effective HBV vaccine in patients that are chronically infected but virally suppressed with NUCs. It is plausible that these B cells are ready to be rejuvenated and respond to HBV antigens with effective long-lasting antibody responses by intervention/s that target the inhibitory pathways. Recent back to back studies demonstrated partial rescue of HBs specific B cells in CHB by PD-1 blocking^41,42^ indicating the defect is not permanent. Future HBV cure strategies for circumventing this block to seroconversion could test synergistic blocking of inhibitory PD-1 and FcRL pathways.

## Methods

### Patient samples

Peripheral blood mononuclear cells (PBMC) were isolated from whole blood by density-gradient centrifugation from CHB patients and healthy controls. Cohort 1 patients were HBV mono-infected (n = 18) and not undergoing any treatment. We also tested another group of patients (cohort 2, HBV mono-infected, n = 6 and HBV/HIV co-infected, n = 10) from whom samples were available from baseline and at approximately week 85–96 of adefovir therapy. Patient characteristics and clinical trial details for HBV or HIV/HBV infected patients that received adefovir treatment, were published previously^43^. All patients remained HBs antigen positive at the end of treatment time-point. The control comparator group consisted of HBV-uninfected individuals (n = 20). Clinical and demographic characteristics of all comparator groups are provided in Table 1. Fine needle liver biopsies were available from a subset of HBV patients (under approved IRB protocols); liver infiltrating lymphocytes were isolated from these biopsies using methods described previously^44^.

**Table 1:** Demographic and clinical characteristics of chronic HBV and comparator groups.

Characteristic		Chronic HBV	Chronic HBV Cohort 2	Healthy controls
		Cohort 1	(Pre and post-Adefovir groups)	
Subjects		18	16	20
Gender, M(F)		12(6)	11(5)	10(8)
Age, median yrs. (range)		42.1(18–70)	38.5(17–71)	35.1(24–65)
Race	Asians	6	2	4
	Africans	10	0	1
	African Americans	1	2	5
	Caucasians	1	12	8
	Others	0	0	0
ALT, median U/ml (range)		26(14–100)	—	NA
Baseline viremia, IU/ml, median (range)		71963	3.9 × 10^9^	NA
		(245–4.5 × 10^6^ IU/ml)	(7660–1.14 × 10^11^ IU/ml)	
HBeAg+		1	16	NA
HIV+		0	11	0

### Ethics statement

Samples used here were from already existing collection (clinical trial NCT00023153). All participants signed informed consent approved by the National Institute of Allergy and Infectious Disease Institutional Review Board at the time of screening and enrollment and all samples were anonymized. All methods utilized for this study were performed in accordance with the relevant guidelines and regulations.

### Antibodies and flow cytometry

Polychromatic flow cytometry was used to analyze various B and Tfh cell subsets. Multiple Tfh subsets were determined using monoclonal antibodies including: CD3 Alexa Fluor 700, CD4 PE-Cy7, CD45RA BV605, CCR7 BV510, CXCR5 BV421, CCR6 FITC, CXCR3 APC, PD-1 PerCPCy5.5, ICOS PE. For B cell phenotyping, the following antibodies were used: CD19 FITC, CD20 BV421, CD10 PE, CD21 PE-Cy7, CD27 APC, CD24 BV650, CD38 PerCPCY5.5, CD3 APC-Cy7, FcRL4 PerCpCy5.5, FcRL5 PE, PD-1 BV421 (from Biolegend, eBiosciences or BD biosciences). All panels included staining with Live/Dead Cell viability stain (Invitrogen, California, USA) followed by staining with combinations of antibodies at 4 °C. Stained cells were washed with PBS and fixed in 1% paraformaldehyde till data acquisition on FacsAria. Data were analyzed using FlowJo (Treestar Oregon, USA).

### Tfh IL-21 and CD40L production

CD40L expressed on Tfh interacts with CD40 expressed on B cells in order to provide help to and participate in B cell differentiation and generation of plasma cells. For assessing function of Tfh in chronic patients, PBMC were stimulated with PMA (10 ng/ml), Ionomycin (1 μg/ml), αCD28 (2 μg/ml), αCD29 (1 μg/ml), Brefeldin A (10 μg/ml) and Golgistop for 4 hours as described previously^45^. Following staining for surface markers CD3, CD4, CXCR5, Live/dead at 4 °C for 30 minutes cells were fixed and permeabilized using BD cytofix cytoperm kit and stained with antibodies against IL-21 and CD40L at 4 °C for 30 minutes. Cells were acquired on FacsAria and data analyzed with FlowJo (Tree Star).

### B cell ELISPOT assay

HBs antigen specific B cell response was tested with ELISPOT in CHB (n = 14) and healthy individuals with history of HBV vaccination (n = 14). In order to investigate compartmentalized HBs antigen specific B cell response, we sorted naïve (CD19+ CD21hiCD27−), classical memory (CD19+ CD21+ CD27+) and atypical memory B (CD19+ CD21-CD27−) B cells on FACS aria from CHB (N = 3) and individuals with history of HBV vaccination (N = 3). For *ex vivo* ELISPOTs, vaccinated samples were obtained from individuals one week after last booster. For memory B cell ELISPOT, unfractionated or sorted B cell subsets were stimulated for 5 days following which B cell ELISPOT was conducted. Briefly, frozen PBMC were thawed and rested overnight. Cells were stimulated with R-848 (1 μg/ml) and IL-2 (10 ng/ml) for 5 days at 37 °C to aid memory B cell differentiation^18,19^. 96 well ELISPOT plates (MabTech) with PVDF membrane were wet with 70% ethanol, washed in ddH2O and pat dried. Wells were coated with anti-human IgG or rHBs antigen (10 μg/ml) overnight at 4 °C. Plates were washed and following blocking with BSA, specific number of live cells were serially diluted and added to wells and incubated at 37 °C for 18 hours. Following washing, biotinylated anti-IgG was added to wells, and detection done with streptavidin alkaline phosphatase and vector blue substrate system. Plates were scanned on ELISpot analyzer for spot counting. HBs specific B cell frequency was calculated by subtracting number of spots counted in negative control wells and data is represented as number of spots/10^6^ cells.

### RNAseq and bioinformatics analyses

To obtain an overview of B cell pathways dysregulated in CHB, CD19^+^CD3^−^ cells were sorted from HBV monoinfected or control samples (n = 5 each) on FacsAria to >98% purity. The HBV patients used here were not on treatment and their characteristics are presented in Table 2. Between 250,000–1,000,000 sorted B cells were collected in RNA later and stored at −80C till processing for RNA extraction. Total RNA was isolated using miRVana kit (Ambion) with manufacturer’s instructions. Whole transcriptome libraries were constructed for sequencing on the Illumina platform using the NEBNext® Ultra™ Directional RNA Library Prep Kit (New England Biolabs, Ipswich, MA). Detailed library preparation, sequencing and analysis methods are provided in Supplementary file). RNAseq analysis was carried out by the Informatics Resource Center, Institute for Genome Sciences, UMDSOM. The DESeq package (v1.10.1) was used to estimate dispersion, normalize read counts by library size to generate the counts per million for each gene, and determine differentially expressed genes between the HBV+ and HBV− samples. Differentially expressed transcripts with a FDR ≤ 0.05 and log_2_ fold change ≥1.5 were used for downstream analyses. Normalized read counts were used to compute the correlation between replicates for the same condition and compute the principal component analysis for all samples. The list of differentially expressed genes was used to compute the enrichment of biological pathways using Ingenuity Pathway Analysis (IPA).

**Table 2:** Patients and controls used for RNAseq.

Characteristic		Chronic HBV (n = 5)	Healthy controls (n = 5)
Age (yrs)		31, 32, 38, 54, 65	29, 31, 33, 49, 58
Race	Asians	3	2
	African Americans	1	2
	Caucacian	1	1
HBeAg+		0	0
HIV+		0	0

### BCR sequencing

Effect of long-term treatment on B cell receptor repertoire was tested by sequencing BCR IgH chains. B-cell receptors were sequenced at high-throughput using bias-controlled multiplex PCR amplification from DNA isolated from PBMC of CHB patients (paired samples before and after treatment) by Adaptive biotechnologies (Seattle, USA). Data was analyzed using immunoSeq Analyzer 3.0 (Adaptive biotechnologies).

### B cell stimulation assays

Having observed an increased CD40L expression from Tfh in chronic patients we sought to determine the consequence of persistent CD40 signaling on B cell phenotypes. Since CHB infection is characterized by high levels of HBV antigens (specifically HBsAg) in patient serum, we tested the consequence of antigen mediated signaling and CD40 ligation on B cell differentiation phenotypes. For this, PBMC were stimulated with a combination of F(ab’)2 (Jackson ImmunoResearch Laboratories, USA), HBV antigens (Fitzgerald Industries International) and CD40L (Jena Bioscience GmbH, Germany). 1 × 10^6^ cells/ml were incubated with anti-IgM/G/A F(ab’)2 (2.5 μg/ml), soluble CD40L (500 ng/ml), HBs Ag (3 μg/ml) or HBc Ag (4 μg/ml) for 3 days. Phenotypes of B cells were evaluated with antibody staining and flow cytometry.

### HBV core binding assay

PBMC were stained with biotinylated HBV core protein in HEPES buffered saline (HBS). The biotinylated HB core protein used for this assay was a kind gift from Gilead Sciences, Inc. Cells were pre-blocked with human IgG and mouse IgG (5 μg per 1 × 10^6^ cells) to block Fc receptors. 1 × 10^6^ cells were incubated with HBV core along with antibody against CD19 FITC for 30 minutes on ice. Cells were washed in staining buffer three times, followed by staining with Avidin PE or anti-biotin PE antibodies. Cells were washed in staining buffer and fixed in 1% paraformaldehyde solution. To determine specificity of HBc binding, competition assay with unconjugated HBc was performed. For this, cells were first incubated with unconjugated HBc to occupy HBc binding receptors followed by washing to remove unbound HBc. Biotinylated HBc was then added as above followed by detection with anti-biotin or avidin PE.

### B cell proliferation

For testing effect of HBcore binding on B cell proliferation, PBMC were labeled with CFSE (5-(and-6-) carboxyfluorescein diacetate succinimidyl ester) using the CellTrace cell proliferation kit using the manufacturer’s instructions (Invitrogen). After washing three times in RPMI medium (10% FBS), cells were incubated with F(ab)2′ or R-848 in presence or absence of HBV core for 4 days at 37 °C. Cells were stained with CD19, CD27 and CD21 and FcRL5 and proliferating B cell subsets measured by dilution of CFSE on gated cells.

### Data analysis

Data were analyzed using GraphPad prism v5.0. For analyses of cellular phenotypes and function, comparison was made between HBV mono-infected patients and healthy controls using t-test or Mann Whitney test for parametric and non-parametric data, respectively. For analyzing the effect of long-term adefovir treatment, before and after treatment samples from same patients (either HBV mono-infected or HIV/HBV co-infected) were analyzed using Paired t-test or Wilcoxon matched-pairs signed rank test. No significant differences were found for most of the observations reported here between HBV mono-infected or HIV/HBV co-infected groups. Hence, data from the two groups of HBV infected treated patients are combined for pre and post treatment analyses presented here, unless noted otherwise.

## Electronic supplementary material

Supplementary information

## Data Availability

All data generated or analyzed during this study are included in this published article (and its Supplementary Information files).
