# Supplementary material for: HBV induces inhibitory FcRL receptor on B cells and dysregulates B cell-T follicular helper cell axis
Source: Sci Rep. 2018 Oct 17;8:15296. doi: 10.1038/s41598-018-33719-x (PMC6193006; doi:10.1038/s41598-018-33719-x)
Supplement: Supplementary file 1 — Supplementary information [file 41598_2018_33719_MOESM1_ESM.pdf]

Supplementary information:

HBV induces inhibitory FcRL receptor on B cells and dysregulates B cell-T follicular helper cell axis

Bhawna Poonia, Natarajan Ayithan, Madhuparna Nandi, Henry Masur and Shyam Kottilil

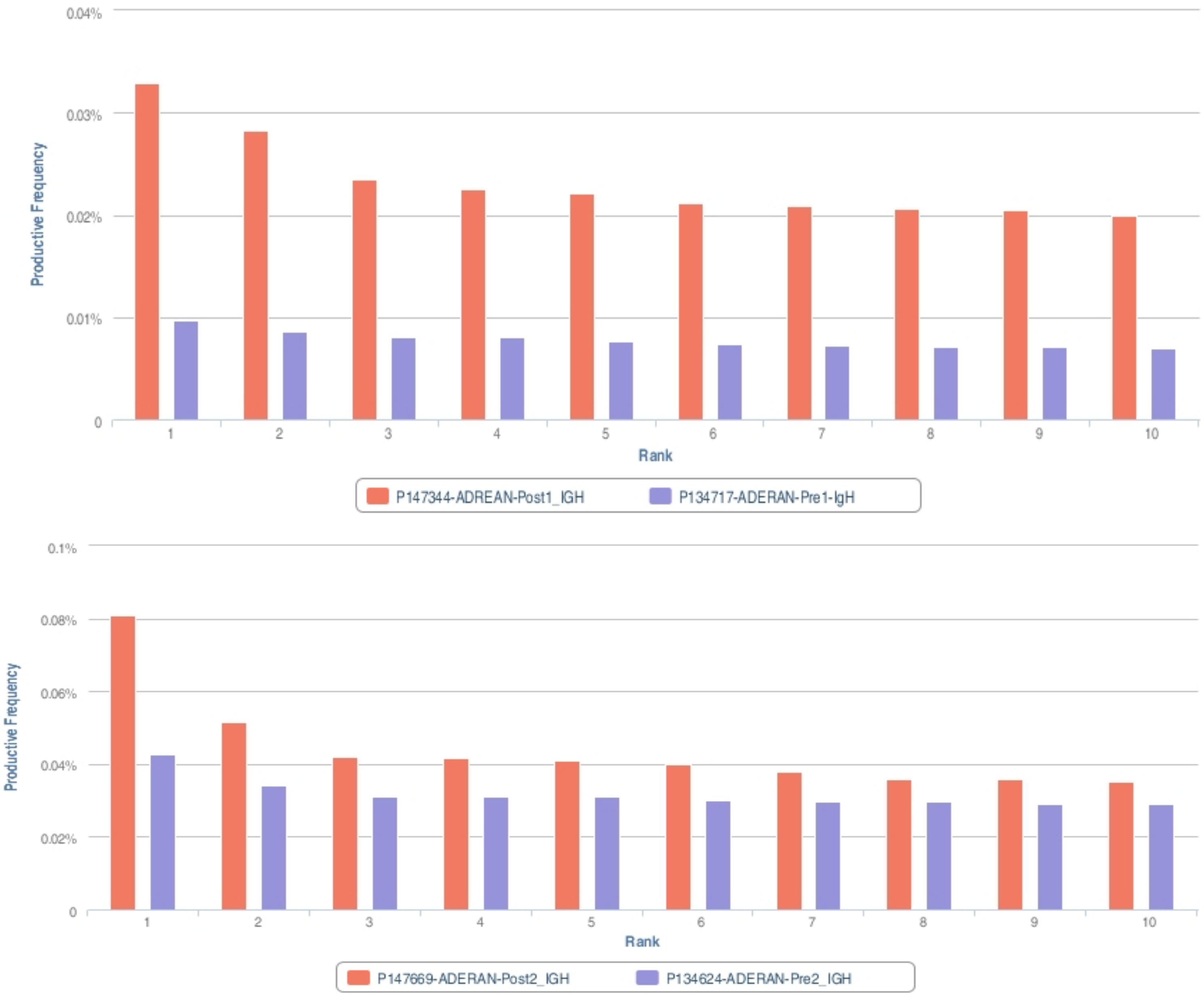

S1: IgH rearrangements before and after NUC therapy. Frequencies of top 10 IgH rearrangements in B cells from 2 CHB patients before (pre, in purple color) or after (post, in red color) NUC therapy. Each patient represents paired sample from individual patient.

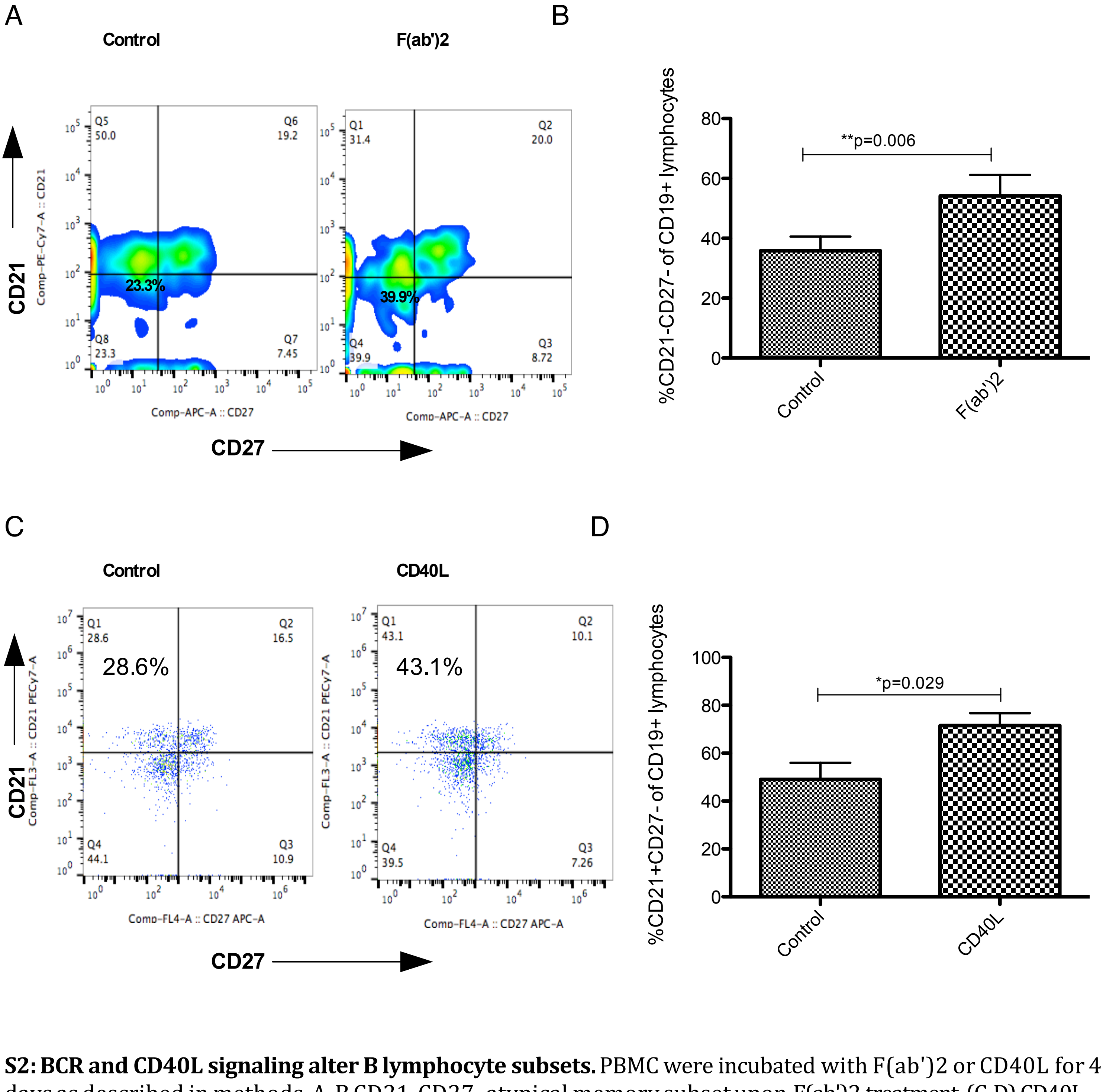

S2: BCR and CD40L signaling alter B lymphocyte subsets. PBMC were incubated with F(ab')2 or CD40L for 4 days as described in methods. A-B CD21-CD27- atypical memory subset upon F(ab')2 treatment. (C-D) CD40L effect on CD21+CD27- naive B cells subset. paired t-test, $p<0.05$ considered significant.

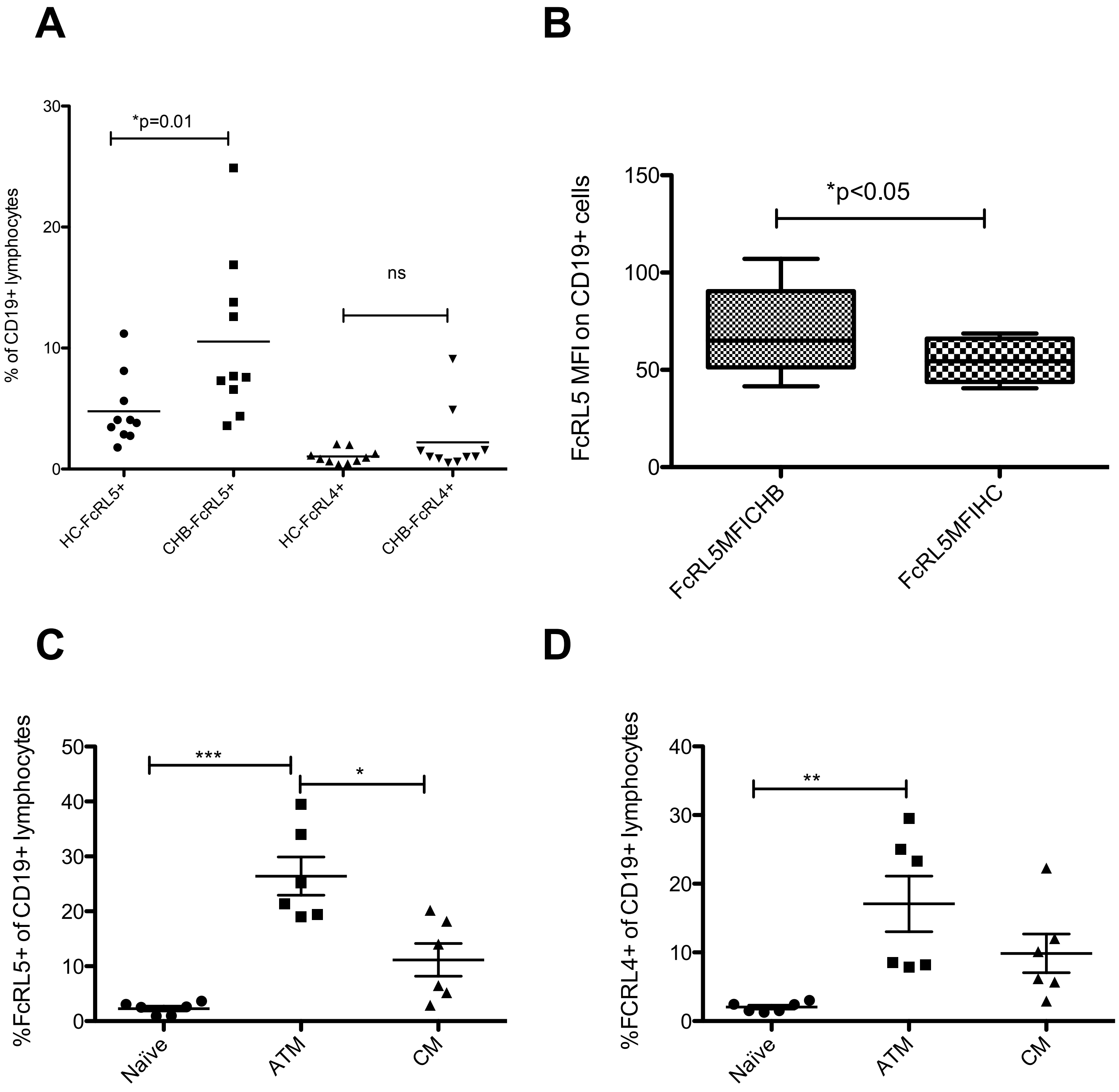

S3. FcRL on atypical memory B cells. (A) Frequency of FcRL5+ and FcRL4+ CD19+ lymphocytes in healthy HC and CHB samples (N=10 each). (B) MFI of FcRL5 on B cells in CHB (FcRL5MFICHB) and healthy (FcRL5MFIHC) (N=10 each). (C) or FcRL4 (D) expressing CD19+ B cells among naïve, atypical memory (ATM) and classical memory (CM) subsets. 1 way ANOVA with turkey's multiple comparison test. * is considered significant (P<0.05)

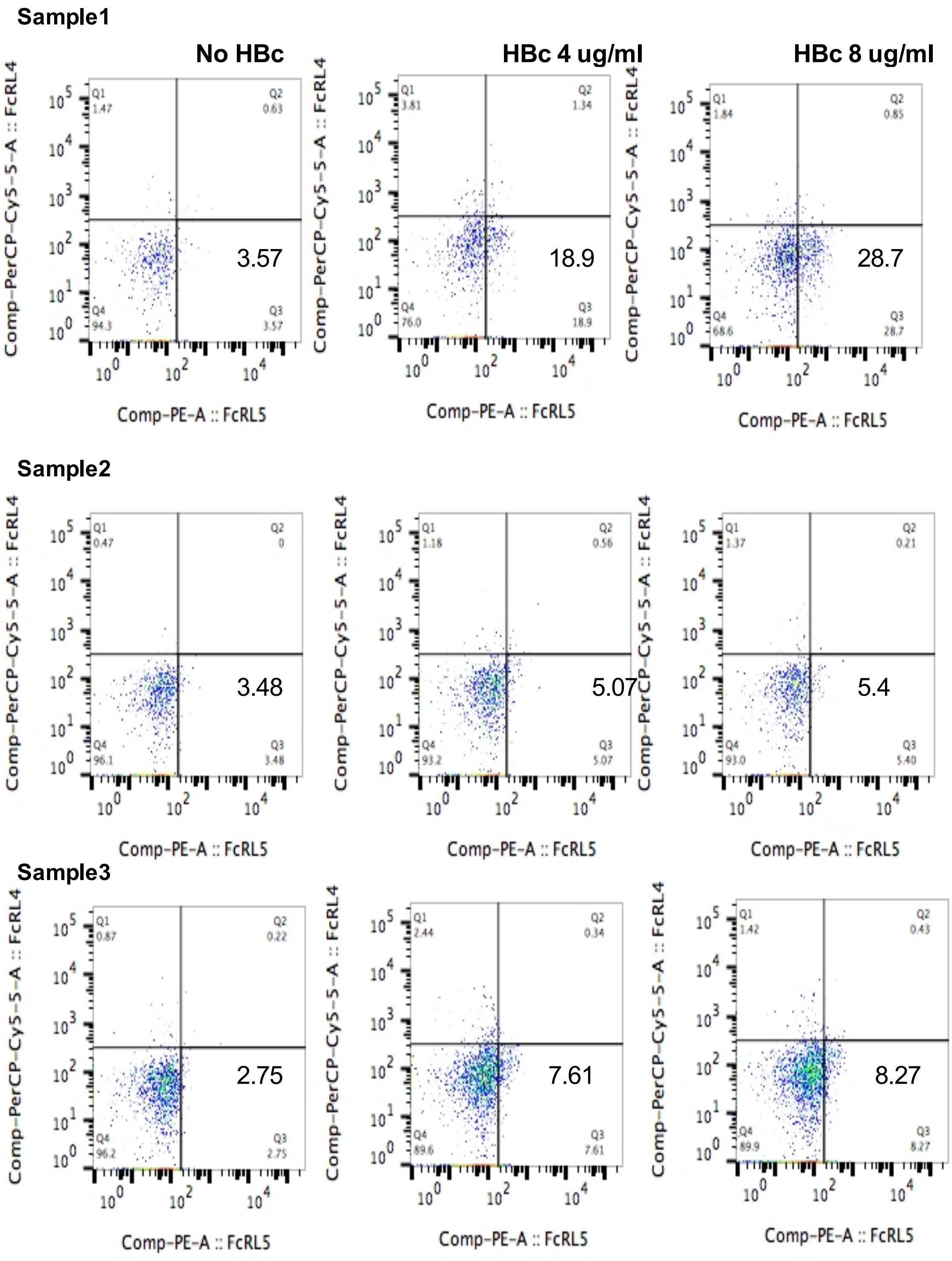

S4. HBc dose dependent FcRL induction. Effect of increasing HBc concentration on FcRL expression on CD19+ lymphocytes in three different samples.

Supplementary table 1: List of DEGs from RNAseq results:

gene_symbol	LFC(pos/neg)
CHADL	-8.398729594
UTS2	8.096201252
TRPC5OS	-7.830271683
RP11-54O7.1	7.748889341
FUT3	-7.638286554
KCNJ5	-7.606280896
PPP1R2P9	-7.379575563
CBARP	-7.327430903
OR7E47P	-7.327207047
HLA-DRB6	-7.275434738
ANKS1B	-7.243971831
HSPA12A	-7.152921017
PTPN21	-7.136323379
LRRC43	-7.088565282
RP4-665J23.4	-7.081909321
NHLRC1	7.078921573
RPL31P47	-6.960441617
SNORD71	-6.874702743
CTB-129P6.7	-6.850349003
CAMK2A	-6.848575093
XIST	-6.827768222
RP11-63N8.3	-6.72084203
PRRC2A	-6.715934187
SEZ6L2	-6.713380555
RP11-665C16.6	-6.710880099
COL2A1	-6.638615955
RP11-548H3.1	-6.624653459
CXCL3	-6.601916208
RP11-167N4.2	-6.596492391
FAM222A-AS1	-6.518205877
AP001062.9	-6.394612247
PRR15	6.381809379
NPHS1	-6.381702028
SPTBN4	-6.334639084
CILP2	-6.327930257
ARSI	-6.309159525
WNT11	6.280179544
RPL18P10	-6.260563679
NCS1	-6.259777844
CTAG2	6.255865835

LMNTD2	6.242667174
RSPH6A	-6.237926732
RPL7AP64	6.231631787
SLC6A12	6.182461264
CACNA1B	-6.098049914
IL1R1	-5.989124064
THSD4	-5.913740306
AC024592.9	-5.800929403
NANOS1	-5.778271289
COL17A1	-5.731554103
PRNCR1	-5.725792687
RP11-1336O20.2	-5.700564882
RP13-580B18.4	-5.683867743
HLA-DRB5	-5.669155339
MAP7D2	-5.629373692
HLA-DRB1	-5.604277166
ADAMTS7	-5.597975861
MTND1P23	5.583031056
SHISA3	5.582710634
XKR4	-5.53577873
SPACA3	5.48696923
COL6A1	-5.476329502
KRT8P34	-5.475856395
CES3	-5.41640662
XXbac-BPG299F13.17	-5.292670409
AC018804.6	-5.203100974
LILRB2	5.164750015
RUNDC3A-AS1	-5.146841541
OLR1	-5.144442406
KIRREL2	-5.052377325
SDC4	-4.923027545
RP4-694B14.8	4.909832417
Y_RNA	-4.877514997
CTD-3028N15.3	-4.828406228
RP13-467H17.1	4.800339357
EDA2R	4.713863391
NGFR	4.681496563
GCSH	4.634019366
CTTN	-4.57567292
HORMAD2-AS1	-4.574986393
IGHA1	4.554473763
TMEM169	4.537086806
ATP9A	-4.522251732

HIST2H4B	4.485746978
MGAT5B	-4.454393855
PDE9A	-4.421475101
ILDR2	-4.420335326
TMEM200A	4.414489743
RP11-327F22.4	-4.389481423
RTN4R	-4.314819169
ANKRD36BP2	4.281124791
RP11-142C4.4	4.280203997
SLC25A24P1	4.274032822
RP11-613H2.2	-4.238343762
FNDC7	-4.179006646
IRF7	4.174967855
PNCK	-4.141197395
RP11-326C3.16	-4.131764436
AC091633.2	-4.114461357
SMO	-4.094007363
SLC16A14	4.059791596
U1	-4.025748039
RP11-446N19.1	3.844182099
BMS1P10	-3.794271425
NLGN4Y	3.792657464
RPS26P21	-3.785651677
HLA-F	-3.76935171
AF127936.9	3.73363588
TNFRSF17	3.702717023
TSIX	-3.697551332
LINC01108	-3.696040003
SEMA3G	3.684202128
IGKV1D-17	3.655389196
RP11-409K20.6	-3.654361984
RP11-359N11.1	-3.652793639
SMAD6	-3.639812376
FAM86JP	3.624893187
AC079325.6	3.624294791
CFAP43	-3.61634866
RRM2	3.616292782
LINC01619	-3.594742365
PTPRU	-3.593935291
RP11-557C18.3	-3.57271293
SDC3	-3.560681992
MIR223	3.54757001
EPB41L4B	-3.526532162

AVP	-3.516625115
SOS1-IT1	3.507477434
F2RL3	3.504465687
FCRLA	3.492549794
RUNDC3A	-3.484222187
LYNX1	3.477260577
HLA-C	-3.474010129
LAMP5	3.465707015
TMEM198	3.436287786
RP5-943J3.2	3.426098078
POU4F1	3.422541271
FUT5	-3.397806009
SERPINI1	3.396156343
FCRLB	3.391454212
SIGLEC6	3.345025204
RPS4Y2	3.335039321
PLEKHS1	-3.329916657
AC007050.17	-3.323877487
RP11-307E17.8	3.296630802
SPN	3.281122454
CKMT2-AS1	3.252598021
PDE2A	-3.240165299
MTATP8P2	-3.237806843
APLP1	-3.227713483
SLCO4A1	-3.22255735
AC066692.3	-3.213607357
CLLU1OS	3.175779901
RP11-432J24.3	-3.164723853
OR2W3	3.154127807
RN7SL34P	-3.15350888
LINC00865	3.153195387
DNAH2	-3.131727572
NRIP3	-3.122691521
SDC1	3.101400069
SIRPB2	3.099146904
COL1A1	-3.09801219
RNU6-30P	-3.0927209
TFCP2L1	-3.085545104
PI4KAP1	-3.081679585
CCR2	3.062482167
TMSB4Y	3.060361317
TNFAIP8L2	3.057808683
RP11-677M14.3	-3.009895079

HSD11B2	-3.007027139
RP11-266L9.5	2.997734769
TCL1A	2.988738736
LZTS3	-2.98785227
RP11-219E7.1	2.977388083
ADRA2B	-2.965263508
AF127936.5	2.964108935
CCDC74B	-2.959245762
CTRC	-2.955874877
CNTD2	-2.951023731
RGPD2	-2.943845149
BANF1P2	2.934027274
LINC00278	2.9310402
RGPD1	-2.923062277
GRK7	-2.916645382
HCRTR1	-2.909495252
GCM1	-2.908927849
CMPK2	2.895080119
GLYATL1P3	2.885724011
CTD-2542L18.1	-2.881367845
CX3CR1	2.876144784
U3	-2.875104754
IFI44L	2.844289494
NINJ1	-2.843163574
ALDH1B1	2.838399115
RPP25	2.837338037
DOCK4	-2.823089007
IGFALS	-2.822491825
CTD-2270L9.4	-2.819577236
ARV1	2.812998559
RP11-727F15.13	-2.811106902
RP11-66H6.4	-2.806353455
HTR2B	-2.795203935
TST	2.791365636
LMNA	-2.783131873
BIRC5	2.781372051
AOC3	-2.781304764
SELPLG	2.776041714
EREG	-2.775448668
IGKV1D-16	2.770608784
LINC01504	2.767469341
RP11-1191J2.5	2.76432261
BHLHA15	2.741449522

BAMBI	-2.739123171
CDCA5	2.73829338
ZNF442	2.738273535
FGFBP3	2.722569862
VWCE	-2.715079186
RPL31P11	2.712018637
GAREM2	2.708245161
SAMD9L	2.706371633
AC116366.6	-2.698551746
RP11-325P15.1	-2.696727594
DUSP4	-2.691066235
RP11-259N19.1	2.69070574
AC004556.1	2.68525412
TUBB4A	2.683128359
RGN	-2.679314257
C10orf91	-2.678640831
ZNF441	2.678192424
MKI67	2.676741149
SLC1A2	-2.676279012
RP11-108M9.4	2.664475271
TMEM182	2.657216536
SNX9	-2.655951887
HLA-DQB1-AS1	-2.654923886
ZNF391	2.640860125
FSCN3	-2.632634244
SEMA7A	-2.625125304
SDHAF3	2.618558086
ID1	-2.61170108
PLA2G2D	2.607290766
FADS1	-2.601499441
ERCC6L	-2.599347117
LSMEM1	-2.5984524
IGLV9-49	2.596479506
DSCAML1	-2.596225607
APOE	-2.584961529
SMAD7	-2.578720977
AC010240.2	-2.57687593
C19orf45	-2.573725663
RP11-93B14.10	2.567279786
AC005387.3	-2.563253411
NECTIN2	-2.559872613
RP11-1398P2.1	2.556002776
KIAA0895	-2.555235966

GVINP1	2.546224461
RP11-642A1.1	-2.533920886
LHX3	-2.529421585
PDE4A	-2.526408861
OAS1	2.523198507
CRABP2	-2.518326247
MN1	-2.516132032
VMO1	2.503383851
CTD-3222D19.12	-2.500787442
CCDC102B	2.493511524
MIXL1	2.492478587
N6AMT1	2.491965919
GPRC5D	2.491667976
SDCBP2	-2.487939073
NLGN3	-2.480689192
BACH1-IT2	-2.477379962
Y_RNA	-2.476258055
ATF3	-2.474904395
JAG2	-2.473110647
CD24	2.465173688
RMI2	2.463337218
ZWINT	2.458278914
PLXNA2	-2.453988525
TLR10	2.452386529
ICMT	2.448537117
C9orf131	-2.447286432
NAV2	-2.442014745
SELL	2.44190134
RP11-468E2.10	-2.439659202
RP11-372K14.2	-2.43758958
PDIA4	2.437299718
C1orf116	-2.437063765
ZNF823	2.435775365
SSR4P1	2.426186907
FCGR3A	2.410524162
TCTE1	-2.40809723
PKMYT1	2.405178313
EAF2	2.404488401
CYP1B1	2.400676764
RP11-109L13.1	2.398127943
ALDH6A1	2.396857703
TRG-AS1	2.395262293
RHBDL1	-2.394049312

RP11-228M15.1	2.393442756
MAP10	2.391613333
CDPF1	2.389748205
ZNF860	2.389651443
FAM86C1	2.38720862
FAM222A	-2.385953104
SSPN	2.38455478
TLE6	2.383223028
ABCD2	2.382367788
PHGDH	2.380707267
THBS1	-2.379994434
VIL1	-2.365934006
CARD8-AS1	2.362989791
ADORA2B	2.357242506
SEMA3B	-2.354409499
ACSL1	-2.353512197
CTC-378H22.2	2.352918115
ZNF57	2.351554637
CYSLTR1	2.350527923
FMO4	2.349499045
DHFR2	2.344150266
METTL7A	2.335148886
GGACT	2.332427534
MT1XP1	-2.329292832
KCNH4	-2.327033072
SPINK4	-2.326454538
CAV1	2.326262841
CADM1	2.322473763
AXIN2	-2.321246034
CTB-134F13.1	-2.320632434
PGAP1	-2.317141844
AC079922.3	2.316426492
RP11-67L2.2	-2.314975311
RP11-831H9.3	-2.311510618
GIN1	2.305212177
HRASLS2	2.30417312
CD1C	2.301349704
LMOD2	-2.300090595
DCAF4L1	-2.297901951
LRRC56	2.29770638
RSAD2	2.297669015
RAP1GAP	-2.290021718
HMGB2	2.287785827

CENPQ	2.287366755
RP11-458J1.1	2.277172785
RP11-151N17.1	-2.273576146
VPREB3	2.27258207
LRRN1	2.271779642
HSPE1P26	-2.27130275
ANXA4	2.270852656
GALK1	2.268511492
RP11-1099M24.6	-2.267476988
UGDH	2.254849029
DCLK2	-2.25246375
RP11-432J24.2	-2.250115732
IGKV1D-13	2.249882092
SCIMP	2.248011792
CTD-3126B10.2	-2.246971266
GCNT1	2.244881305
CGNL1	-2.242229214
RP11-88E10.5	2.240048533
HLA-DQB1	-2.237768378
XAF1	2.23495273
IGLC7	2.232399907
CFAP20	-2.231390081
CD24P4	2.230274351
CHPF	2.228697903
AP1S1	2.225076577
S1PR2	-2.216173244
RP1-149A16.3	-2.211970309
LINC01013	2.210227616
CFAP45	-2.20846871
RP11-314N13.9	-2.207768633
NPM1P26	-2.206627925
CDHR3	2.202507212
IRF2	2.202395938
NCEH1	2.197905398
MIR4645	2.195975283
TDRKH	2.194984946
USP18	2.184123218
IGLV8-61	2.182625273
OLFML2A	2.182349582
CD9	2.180663869
G0S2	-2.179084864
MILR1	2.175102644
CCDC34	2.170552828

LINC00921	2.169008445
RP11-182N22.9	-2.165757686
CTD-2528L19.6	2.165453221
GPM6A	2.164223133
DLGAP1-AS1	2.163726543
JHDM1D-AS1	2.160734638
DDR2	-2.15771597
C9orf139	2.145848452
RP5-1021I20.5	-2.14528732
AQP3	2.144864699
JCHAIN	2.144244587
LCN10	2.140191808
RP11-51F16.1	2.138226545
PAM	-2.136035466
DIRC2	-2.135723425
CPNE5	2.135305626
C10orf111	-2.133594832
ZYG11A	-2.129476051
RGPD3	-2.126115852
ZNF691	2.125136382
PAQR8	2.124832448
BMP3	2.124121766
PTAFR	2.121818442
AC018755.16	2.118306528
IGKV3D-20	2.114484948
LBH	2.114318206
KCNK7	-2.113571341
BBS10	2.113325443
PLD4	2.112376252
SLC16A6	2.111548962
PYCR1	2.104575243
ASPM	2.10069187
MPEG1	2.094895918
NDUFV2	-2.094776032
IGLV3-10	2.092243452
Y_RNA	-2.088156497
AC093673.5	-2.080962704
RP11-46C24.7	2.077278693
SENP3-EIF4A1	-2.071464631
SLC4A11	-2.071452949
PPCDC	2.069926281
RAB30-AS1	2.066846691
SMIM14	2.063937124

HIST1H2AC	2.054491195
RAB36	2.054396661
RP11-456P18.2	-2.049060478
PBX4	-2.048169775
C9orf69	2.04546009
MT2A	2.042528228
GCSHP5	2.041257068
CAMKK1	-2.037968244
CEACAM1	2.036915112
CTD-2353F22.1	2.03482269
KLHDC7B	2.034510577
ZNF219	-2.032912922
IGLV3-9	2.031444782
DPYSL2	2.029909107
MKKS	2.028266866
GRIK3	2.026031512
TREML2	2.024366141
BEND3P1	2.022798205
DUSP10	-2.020511127
IL32	2.020011466
TEX14	-2.018617374
MAPK8IP1	-2.017967691
NFKBIE	-2.015909341
VWA5A	2.012249431
LAMB1	-2.010476484
TLR4	2.008211213
CNIH2	-2.007280458
IGKV4-1	1.999546837
RUVBL1	1.998512663
TNFRSF4	-1.996299007
FAM57A	-1.995018995
RP13-554M15.2	-1.992338589
FUOM	1.992149096
NARF-IT1	-1.990318711
KIF11	1.990314699
AP003068.23	1.988962943
SLAMF6	1.988077624
RP11-542H15.1	-1.9878778
IGLV3-27	1.985275105
RP1-149A16.17	-1.983146974
CAT	1.983104564
SLC9A5	-1.98133119
DBP	1.980126376

RPP25L	1.977775829
ZNF594	1.977076275
PIGK	1.973779971
RIC8B	1.972560563
ALMS1-IT1	-1.972523367
AIM2	1.96498274
HHLA2	-1.963795215
FHL2	-1.96371169
HGH1	1.962833955
CTC-378H22.1	1.962521674
RNASEL	1.962019479
PCDH9	1.960062031
ANXA2P2	1.959550416
PPP1R14A	1.958915656
RP11-573G6.4	1.958548307
ZNF696	1.954837994
SNX18	1.954020362
RP11-93B14.9	1.953846011
NUP58	-1.953763364
RP11-143J12.3	-1.95208953
SLC44A5	-1.948160091
EIF1AY	1.944110899
BANF1P3	1.940037432
DNM1P47	1.936785137
SAMHD1	1.935511187
FAM132A	-1.933701594
CD300A	1.933621416
CCDC152	1.919005046
KIAA0040	1.917536448
ABCB9	1.917137024
ALPL	-1.91690492
FCRL3	1.306364327

Supplementary table 2: IPA canonical pathway analysis between HBV negative and positive B cells shows differentially enriched pathways

Ingenuity Canonical Pathways	-log(p-value)	Ratio	z-score	Molecules
Dendritic Cell Maturation	5.22	0.165	-2.646	RELA,PIK3CA,ICAM1,HLA-A,NFKBIE,IL32,HLA-DQA1,IL6,HLA-DQB1,NFKB1,PIK3R4,FCGR2B,HLA-DRB1,NFKBIA,DDR2,NGFR,LY75,STAT1,CD1C,FCGR3A/FCGR3B,COL2A1,NFKB2,STAT4,TLR4,COL1A1,FSCN3,HLA-C,MAPK10,CD86,HLA-DRB5
OX40 Signaling Pathway	4.96	0.259	-1.134	RELA,TNFRSF4,HLA-A,NFKBIE,HLA-DQA1,HLA-DQB1,NFKB2,NFKB1,NFKBIA,HLA-DRB1,HLA-C,MAPK10,HLA-F,HLA-DRB5
Type I Diabetes Mellitus Signaling	4.54	0.189	-1.732	RELA,HLA-A,NFKBIE,HLA-DQA1,IFNGR1,IL1R1,HLA-DQB1,NFKB2,NFKB1,TRADD,HLA-DRB1,CASP9,NFKBIA,HLA-C,NGFR,MAPK10,CD86,STAT1,HLA-F,HLA-DRB5
T Helper Cell Differentiation	4.28	0.217	#NUM!	IL6ST,HLA-A,IL6R,HLA-DQA1,IFNGR1,HLA-DQB1,IL6,STAT4,HLA-DRB1,TGFB1,NGFR,CD86,STAT1,HLA-DRB5,ICOSLG/LOC102723996
Interferon Signaling	3.98	0.278	2.53	RELA,IFIT3,OAS1,MX1,IFI6,IFI35,IFNGR1,STAT1,IFITM2,ISG15
CD28 Signaling in T Helper Cells	3.91	0.167	-1.069	RELA,PTPN6,PIK3CA,ARPC1B,HLA-A,ARPC5L,CSK,NFKBIE,ARPC5,HLA-DQA1,HLA-DQB1,NFKB2,NFKB1,PIK3R4,LCK,HLA-DRB1,NFKBIA,GRAP2,MAPK10,CD86,HLA-DRB5
Altered T Cell and B Cell Signaling in Rheumatoid	3.86	0.193	#NUM!	TLR4,RELA,TLR10,HLA-DRB1,CD79B,HLA-A,TGFB1,TLR6,HLA-DQA1,CD86,NFKB2,IL6,HLA-DQB1,NFKB1,HLA-DRB5,TNFRSF17
Pancreatic Adenocarcinoma Signaling	3.86	0.169	-0.471	RELA,PIK3CA,PLD3,CDK4,HBEGF,KRAS,NFKB2,NFKB1,PIK3R4,RALBP1,BIRC5,SIN3A,PLD4,HMOX1,CASP9,TGFB1,CDKN1A,MAPK10,STAT1,CDK2
TREM1 Signaling	3.63	0.2	-0.535	ITGB1,CXCL3,STAT5A,TLR4,RELA,CXCL8,TLR10,ICAM1,TLR6,CD86,NFKB2,IL6,FCGR2B,NFKB1
Crosstalk between Dendritic Cells and Natural Kill	3.5	0.18	#NUM!	RELA,HLA-A,CD69,TNFSF10,IL6,NFKB2,NFKB1,FSCN3,TLR4,CAMK2A,HLA-DRB1,HLA-C,CD86,HLA-F,HLA-DRB5,NECTIN2
B Cell Development	3.26	0.276	#NUM!	HLA-DRB1,SPN,CD79B,HLA-A,HLA-DQA1,CD86,HLA-DQB1,HLA-DRB5
Role of NFAT in Regulation of the Immune Respor	3.25	0.139	-1.606	BLNK,RELA,PIK3CA,HLA-A,NFKBIE,HLA-DQA1,CSNK1A1,KRAS,HLA-DQB1,NFKB1,PIK3R4,FCGR2B,LCK,HLA-DRB1,NFKBIA,XPO1,GNG5,FCGR3A/FCGR3B,CD79B,GNA12,CSNK1D,NFKB2,MEF2D,CD86,HLA-DRB5
Activation of IRF by Cytosolic Pattern Recognition	3.19	0.2	0	DHX58,RELA,IRF7,NFKBIA,NFKBIE,ZBP1,MAPK10,IL6,NFKB2,STAT1,NFKB1,ISG15
iCOS-iCOSL Signaling in T Helper Cells	3.01	0.154	-3	GAB2,RELA,PIK3CA,HLA-A,CSK,NFKBIE,HLA-DQA1,HLA-DQB1,NFKB2,NFKB1,PIK3R4,LCK,HLA-DRB1,NFKBIA,CAMK2A,GRAP2,HLA-DRB5,ICOSLG/LOC102723996
Role of JAK family kinases in IL-6-type Cytokine Si	2.96	0.28	#NUM!	IL6ST,STAT5A,IL6R,MAPK10,OSM,IL6,STAT1
Phenylethylamine Degradation I	2.92	0.75	#NUM!	AOC3,AOC2,ALDH3A2
Th1 and Th2 Activation Pathway	2.9	0.133	#NUM!	STAT5A,PIK3CA,ICAM1,TNFRSF4,JAG2,BHLHE41,CHD4,HLA-A,IKZF1,IL6R,HLA-DQA1,IFNGR1,HLA-DQB1,IL6,NFKB1,PIK3R4,IL24,STAT4,HLA-DRB1,TGFB1,CD86,STAT1,HLA-DRB5,ICOSLG/LOC102723996
TWEAK Signaling	2.86	0.242	0	RELA,TRADD,NFKBIA,CASP9,NFKBIE,NFKB2,NFKB1,BAG4
IL-8 Signaling	2.72	0.128	-0.816	RAC2,RELA,PIK3CA,ICAM1,KRAS,LIMK2,NFKB1,PIK3R4,EIF4EBP1,BRAF,HMOX1,CYBB,GNG5,CR2,RPS6KB1,SRC,CXCL8,PLD3,RHOC,GNA12,HBEGF,CSTB,PLD4,MAPK10,IRAK2
Aryl Hydrocarbon Receptor Signaling	2.68	0.141	#NUM!	ALDH1B1,SRC,RELA,NQO2,CDK4,SLC35A2,NFKB2,IL6,NFKB1,CYP1B1,TGFB1,ALDH3A2,RARA,CDKN1A,ALDH18A1,ALDH5A1,AHR,ALDH6A1,CDK2
iNOS Signaling	2.67	0.209	-1.667	RELA,TLR4,NFKBIA,NFKBIE,IFNGR1,NFKB2,STAT1,NFKB1,IRAK2
Inflammasome pathway	2.65	0.286	0	CXCL8,TLR4,AIM2,PYCARD,NFKB2,NFKB1
Th2 Pathway	2.65	0.137	-0.577	STAT5A,PIK3CA,ICAM1,TNFRSF4,JAG2,BHLHE41,CHD4,HLA-A,IKZF1,HLA-DQA1,HLA-DQB1,NFKB1,PIK3R4,IL24,STAT4,HLA-DRB1,TGFB1,CD86,HLA-DRB5,ICOSLG/LOC102723996
Rac Signaling	2.64	0.147	-1.5	ITGB1,RPS6KB1,RELA,PIK3CA,ARPC1B,ARPC5L,ARPC5,BRK1,KRAS,LIMK2,NFKB2,NFKB1,PIK3R4,ANK1,ARFIP2,CFL2,CYBB
PKCθ Signaling in T Lymphocytes	2.6	0.142	-2.357	RAC2,RELA,PIK3CA,HLA-A,NFKBIE,HLA-DQA1,KRAS,HLA-DQB1,NFKB2,NFKB1,PIK3R4,LCK,HLA-DRB1,NFKBIA,CAMK2A,GRAP2,CD86,HLA-DRB5
IL-6 Signaling	2.6	0.142	-3.3	IL6ST,RELA,CXCL8,ABCB1,PIK3CA,NFKBIE,IL6R,KRAS,IL1R1,NFKB2,IL6,NFKB1,PIK3R4,COL1A1,NFKBIA,NGFR,MAPK10,MCL1
Graft-versus-Host Disease Signaling	2.59	0.205	#NUM!	HLA-DRB1,HLA-C,HLA-A,HLA-DQA1,CD86,IL6,HLA-DQB1,HLA-F,HLA-DRB5
Communication between Innate and Adaptive Im	2.44	0.159	#NUM!	TLR4,CXCL8,TLR10,HLA-DRB1,HLA-C,HLA-A,TLR6,CD86,IL6,IGHA1,HLA-F,HLA-DRB5,TNFRSF17
Antigen Presentation Pathway	2.44	0.211	#NUM!	HLA-DRB1,HLA-C,HLA-A,HLA-DQA1,CANX,HLA-DQB1,HLA-F,HLA-DRB5
Role of JAK1, JAK2 and TYK2 in Interferon Signalir	2.33	0.25	#NUM!	RELA,PTPN6,IFNGR1,NFKB2,STAT1,NFKB1
Role of PKR in Interferon Induction and Antiviral F	2.3	0.2	#NUM!	RELA,NFKBIA,CASP9,NFKBIE,NFKB2,STAT1,NFKB1,RNASEL
Role of RIG1-like Receptors in Antiviral Innate Imr	2.23	0.195	-0.816	DHX58,RELA,IRF7,NFKBIA,NFKBIE,NFKB2,NFKB1,TRIM25
MIF Regulation of Innate Immunity	2.23	0.195	-1.414	RELA,TLR4,NFKBIA,PLA2G2D,NFKBIE,MAPK10,NFKB2,NFKB1
MIF-mediated Glucocorticoid Regulation	2.22	0.212	-1.134	RELA,TLR4,NFKBIA,PLA2G2D,NFKBIE,NFKB2,NFKB1
Autoimmune Thyroid Disease Signaling	2.17	0.19	#NUM!	HLA-DRB1,HLA-C,HLA-A,HLA-DQA1,CD86,HLA-DQB1,HLA-F,HLA-DRB5
CD27 Signaling in Lymphocytes	2.15	0.176	-1.134	RELA,NFKBIA,CASP9,CD70,NFKBIE,MAPK10,NFKB2,CD27,NFKB1
Erythropoietin Signaling	2.15	0.152	#NUM!	RELA,RPS6KB1,STAT5A,SRC,PTPN6,PIK3CA,NFKBIA,NFKBIE,KRAS,NFKB2,PIK3R4,NFKB1
Role of Pattern Recognition Receptors in Recogni	2.1	0.13	-1.265	CXCL8,RELA,PIK3CA,OAS1,OAS2,IL6,NFKB2,OAS3,NFKB1,PIK3R4,RNASEL,TLR4,IRF7,TGFB1,TLR6,MAPK10,OSM
HMGB1 Signaling	2.1	0.13	-2.5	CXCL8,RELA,PIK3CA,ICAM1,RHOC,IFNGR1,KRAS,IL1R1,IL6,NFKB2,NFKB1,PIK3R4,TLR4,TGFB1,NGFR,MAPK10,OSM

Methods: RNAseq and bioinformatics analysis

First, mRNA was isolated using the NEBNext Poly(A) mRNA Magnetic Isolation Module. Then libraries were prepared using a modified version of manufacturer's protocol. The cDNA was purified between enzymatic reactions with AMPure SPRIselect (Beckman Coulter Genomics, Danvers, MA) and size selection was performed using the AMPure SPRIselect. The PCR amplification step was performed with primers containing an index sequence seven nucleotides in length. Libraries were evaluated using the Gx touch capillary electrophoresis system (Perkin Elmer, Waltham, MA). Libraries were pooled and sequenced using two flowcell lanes on an Illumina HiSeq 4000 150PE run.

RNAseq analysis was carried out by the Informatics Resource Center, Institute for Genome Sciences, UMDSOM. Paired-end Illumina libraries were mapped to the Human reference, Ensembl release GRCh38.86, using TopHat v2.1.1, using default mismatch parameters. Read counts for each annotated gene were calculated using HTSeq. The DESeq package (v1.10.1) was used to estimate dispersion, normalize read counts by library size to generate the counts per million for each gene, and determine differentially expressed genes between the HBV+ and HBV- samples. Differentially expressed transcripts with a FDR ≤ 0.05 and $\log_2$ fold change ≥ 1.5 were used for downstream analyses. Normalized read counts were used to compute the correlation between replicates for the same condition and compute the principal component analysis for all samples. The list of differentially expressed genes was used to compute the enrichment of biological pathways using Ingenuity Pathway Analysis (IPA).
